# Proteomics of the heart

**DOI:** 10.1152/physrev.00026.2023

**Published:** 2024-02-01

**Authors:** Oleg A. Karpov, Aleksandr Stotland, Koen Raedschelders, Blandine Chazarin, Lizhuo Ai, Christopher I. Murray, Jennifer E. Van Eyk

**Affiliations:** Smidt Heart Institute, Advanced Clinical Biosystems Research Institute, Cedars-Sinai Medical Center, Los Angeles, California, United States

**Keywords:** cardiac, cardiovascular, mass spectrometry, posttranslational modifications, proteomics

## Abstract

Mass spectrometry-based proteomics is a sophisticated identification tool specializing in portraying protein dynamics at a molecular level. Proteomics provides biologists with a snapshot of context-dependent protein and proteoform expression, structural conformations, dynamic turnover, and protein-protein interactions. Cardiac proteomics can offer a broader and deeper understanding of the molecular mechanisms that underscore cardiovascular disease, and it is foundational to the development of future therapeutic interventions. This review encapsulates the evolution, current technologies, and future perspectives of proteomic-based mass spectrometry as it applies to the study of the heart. Key technological advancements have allowed researchers to study proteomes at a single-cell level and employ robot-assisted automation systems for enhanced sample preparation techniques, and the increase in fidelity of the mass spectrometers has allowed for the unambiguous identification of numerous dynamic posttranslational modifications. Animal models of cardiovascular disease, ranging from early animal experiments to current sophisticated models of heart failure with preserved ejection fraction, have provided the tools to study a challenging organ in the laboratory. Further technological development will pave the way for the implementation of proteomics even closer within the clinical setting, allowing not only scientists but also patients to benefit from an understanding of protein interplay as it relates to cardiac disease physiology.

## Graphical abstract

**Figure:**
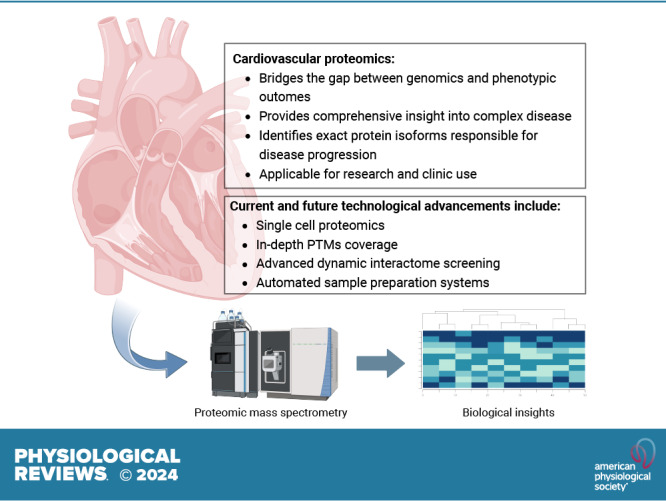

CLINICAL HIGHLIGHTS

- Proteomic-based mass spectrometry (MS) is a powerful tool for identifying changes in protein concentrations within cardiac tissue and the heart.
- Integration of proteomic data with other omics provides for a comprehensive view of intracellular responses to external changes.
- Technological advancements within proteomic mass spectrometry have allowed a higher throughput of samples, an increased dynamic range, and a higher fidelity of data.
- Proteomic applications have long been applied to cardiac research, identifying molecular mechanisms of cardiovascular disease with the help of animal models.
- The proteome can be fractionated into subproteomes, improving acquired data on the mass spectrometer.
- Posttranslational modifications implement a further level of protein regulatory mechanisms, thereby increasing the complexity of protein dynamics.
- Future advancements in the field focus on a higher throughput of samples, single-cell proteome identifications, automation of sample handling, and clinical integrations of MS technologies for use with patients.

## 1. INTRODUCTION

The proteome consists of all expressed proteins within a cell, tissue, or organ and serves as the ultimate end effector of biological functions. Proteins catalyze the chemical reactions that yield the seemingly endless array of biomolecules, glycans, lipids, nucleotides, metabolites, peptides, and proteins that provide scaffolding, nourishment, sustenance, and the dynamic responses to external and internal stimuli on which biology depends (1). Whereas the genome is aptly analogous to an architectural blueprint, the proteome is more than the completed structure. Instead, the proteome is the blueprint dynamically made manifest: a fully staffed and functional factory, constantly attempting to maintain and repair itself while actively receiving, relaying, and responding to internal and external stimuli (2).

Although each protein is translated from its corresponding gene sequences, expression differs in a cell type- and context-dependent manner, not only in terms of relative abundance but also through a multitude of chemical states. These different “proteoform” representations include cotranslational modifications such as posttranslational modifications (PTMs), cleavage products, and amino acid sequence isoforms (3). This chemical complexity endows the proteome with the requisite flexibility to underscore cellular responses to immediate environmental stimuli, to fine-tune differential signaling changes between cell types, and to coordinate metabolic and proliferative processes. The expression of protein quantities and proteoforms provides a molecular snapshot of physiological and disease processes. By extension, proteomics is the quantitative study of a complement of proteins whose expression, structural conformation, and chemical alterations are dependent on biological and temporal context (4, 5).

Cardiac proteomics narrows the focus to the proteins expressed in the heart. Cardiac muscle is a highly specialized tissue composed of many cell types with unique functions, as well as separate proteomes that operate in concert to yield synchronous myocardial excitation-contraction (6). The principal cell type is the cardiomyocyte, which is the fundamental unit and end effector of systole and diastole (7). The coordinated contraction and relaxation of these cells cause blood to circulate throughout the body. Cardiomyocytes are long and cylindrical in shape and contain an array of unique proteins essential to producing contraction while preserving structure (8). Beyond the cardiomyocyte, coordinated contractility depends on the pacemaker cells of the sinoatrial node, the nerve cells within the atrioventricular bundle of His, and Purkinje fibers (9). The high and constant energy demand of cardiomyocytes, along with the rhythmic forces they generate, depend on the many specialized cells of the vascular system and structural chassis, respectively (10). In addition, there are cardiac fibroblasts, pericytes, adipocytes, as well as endothelial, smooth muscle, mesothelial, and various immune cells comprising the complete proteome of the heart (11). Proteomics is a key tool for systematically understanding the context dependence and complexity of this biological system: its hierarchical workflows can serve to differentiate cell types, catalog total protein composition, determine protein complexes and neighborhoods, and translate molecular structure-function relationships to intact cells and tissues.

The heart is prone to a number of life-threatening and widespread diseases including coronary artery disease, heart failure (HF), and arrhythmias (9, 12–17). These diseases represent an altered context within the myocardium and are thus revealed as perturbations to its complex network of regulatory proteins (18). At its most basic level, proteomics allows for more in-depth coverage of a biological process or pathway to reveal interconnections between networks and subproteomes across a cell and across an organ. Thus, baselining the diseased proteome to its healthy counterpart can provide rich datasets whose interpretation reveals increasingly detailed and complex insights into cardiovascular physiology (19, 20). Often, the complexity of proteomics data can be overwhelming. Their interpretation benefits from strong bioinformatics support and an understanding of the current state of biological knowledge such that hypotheses can be developed and new paradigms uncovered (21–23).

### 1.1. Protein Complexity and Proteoforms

**FIGURE 1.**
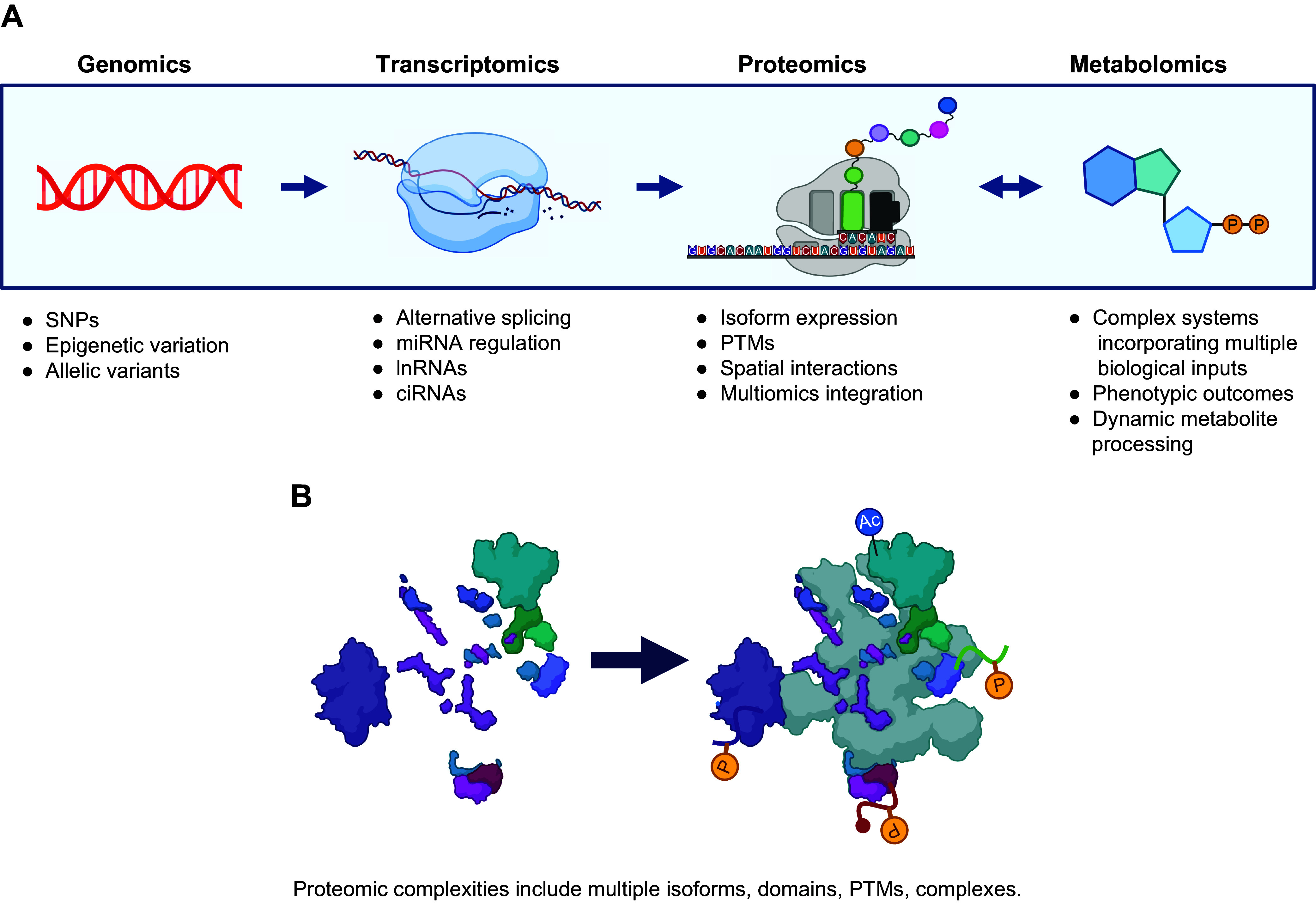
Complexity of data interpretation across the integrated omics. *A*: the central dogma of molecular biology is represented correlating with increasing data complexity. The power of omics data is at its strongest when multiple disciplines are integrated together. Variable factors at each omic interval led to exponential increase of data complexity, and the combination of such data is the exact interface where technological advancements are needed. ciRNA, circular RNA; lnRNA, linear RNA; PTM, posttranslational modification; SNP, single-nucleotide polymorphism. *B*: proteomic complexities arise from multiple proteins of distinct families coming together into a multivariate functional complex, able to be regulated via external activators and inhibitors. Additionally, PTMs can modify the protein complex’s functions, further complicating the dynamic function of protein structures in situ.

In keeping with the central dogma of molecular biology, genetic information within cells is encoded as DNA in the genome, transcribed to RNA intermediates in the transcriptome, and ultimately translated to proteins in the proteome (24) (FIGURE 1*A*). Whereas the genetic sequence of DNA is generally stable, its expression is developmentally and environmentally modified through epigenetic factors. These factors, which underscore cell differentiation, serve to regulate the extent to which genes are transcribed (25). The transcriptome is defined as the complement of RNA transcripts within a cell or tissue, and although transcriptomics provides a first-stage quantitative accounting of gene expression, mRNA translation is highly regulated and therefore the abundance of a protein depends as much on the extent to which its mRNA is translated as it does on the extent to which that protein undergoes degradation (26). For these reasons, quantitative proteomic analysis represents the best and most accurate measure of the end effects of gene expression (27). As well, proteomics including PTMs like methylation can provide insight into transcription and translational regulation. Changes, for example in specific histone deacetylase (HDAC) methylation status, DNA enhancer proteins, ribosome composition, and RNA binding proteins involved in capping and processing of RNA, can provide insights into how the proteome is being regulated. On the basis of these data, additional proteomics experiments focusing on an aspect can be designed and carried out as pointed out by Hasman et al. (28), among others.

Finally, owing to their enzymatic roles in anabolic and catabolic processes, the metabolome, glycome, and lipidome, can be considered a functional end point of the proteome (29). In a broad sense, these highly dynamic biomolecules encompass small molecules, lipids, and complex sugars. Although not directly encoded in the genome, the metabolome nevertheless represents the quantitative sum of all biochemical activities in a cell, and these activities are fundamentally catalyzed and regulated by proteins (30). However, some metabolites provide a feedbacklike mechanism, as they can modify proteins, directly resulting in altered protein function.

Proteins are most fundamentally defined by their amino acid sequence, or primary structure. Owing to the physicochemical properties of specific polypeptide sequences, specific motifs including alpha-helices and beta-sheets form as secondary structures. These motifs exist within the larger framework of a fully translated protein, whose specifically folded conformation is defined as its tertiary structure. Finally, mature proteins often exist as functional associations of several protein subunits, referred to as quaternary structures. Mature proteins are dynamic entities that can undergo conformational changes, and bind other proteins or chemical moieties, and may change their localization within a cell (FIGURE 1*B*). The biological complexity of protein folding and movement throughout the cell is ripe for proteomics analysis, and tools in this space are continuously being developed.

Protein abundances are dynamically regulated through gene expression, relative protein variant/isoform expression, its formation into a protein complex, and subsequent degradation (31). All of these processes ultimately stem from contextual inputs at the cellular level. Additional regulation and modulation of protein activities occur through cotranslational and posttranslational modifications (32). These modifications include phosphorylation, glycosylation, acetylation, methylation, and many others. Most are dynamic in nature, and many occur in combination and/or in competition with one another. The resulting proteoforms add chemical complexity, diversity, and functionality to the proteome. For example, PTMs like methylation can provide insight into transcription and translational regulation. Changes in HDAC methylation status, DNA enhancer proteins, ribosome composition, and RNA binding proteins involved in capping and processing of RNA can impact how the proteome is being regulated (28). Far from being decorative, these cotranslational modifications and PTMs are fundamental responsive elements of cell biology. Proteins are regulated by three primary chemical parameters, size, charge, and hydrophobicity, and these govern their structure and interactions with partner proteins and other moieties (e.g., DNA, RNA, metabolites). By modifying one or more of these three parameters, PTMs can serve to alter the structure and function of a protein. Thus, proteoforms can differ in their structure, function, and stability, thereby leading to altered roles in cellular processes. For example, different proteoforms of a given protein can have different binding partners, interact with different signaling pathways, or serve as an inactive reservoir (33).

## 2. GOAL AND SCOPE

The goal of this review is to provide an overview of the history and current state of the art for how proteomic techniques are applied to research questions on the heart in health and disease. This includes tissue from human and animal model organisms, as well as cell-based models such as cardiac cell-like cell lines and neonatal rat ventricle myocytes, and respective proteomic approaches. Although we have attempted to provide an extensive review, we could not cover all model systems including some cell-based systems like inducible pluripotent stem cells or several large-animal models that have limited proteomic datasets. This review does not include proteomic applications for plasma and body fluid analysis for the discovery of biomarkers or proteomic applications within epidemiology and population sciences.

This review is divided into six sections: *1*) evolution of proteomics of the heart, *2*) current proteomic technologies, *3*) proteomes of the heart and associated pathologies, *4*) subproteomes of the heart, *5*) posttranslational modifications and chemical diversity of proteins, and *6*) future perspectives. Many of these sections include subsections that cover different methods ranging from large-content protein quantification including information about sample preparation, mass spectrometry (MS)-based data acquisition, data analysis, and bioinformatics to more targeted investigations of protein PTMs (e.g., phosphorylation, acetylation, oxidative PTMs, ubiquitin and ubiquitin-like PTMs, citrullination). The review also covers the application of proteomics for biological and pathological insights including human studies of heart failure (HF) and myocardium remodeling, as well as animal models of HF, remodeling, ischemia-reperfusion, and preconditioning. Within this context we highlight different subproteomes such as those obtained from sequential fractions, differential centrifugation, or affinity capture approaches, including protein complexes and several PTMs. Unfortunately, it was not impossible to include all published proteomics-based papers; rather we have included the seminal papers as well as those articles that illustrate a particular point or method pertinent to a specific section of the review.

## 3. EVOLUTION OF PROTEOMICS OF THE HEART

**FIGURE 2.**
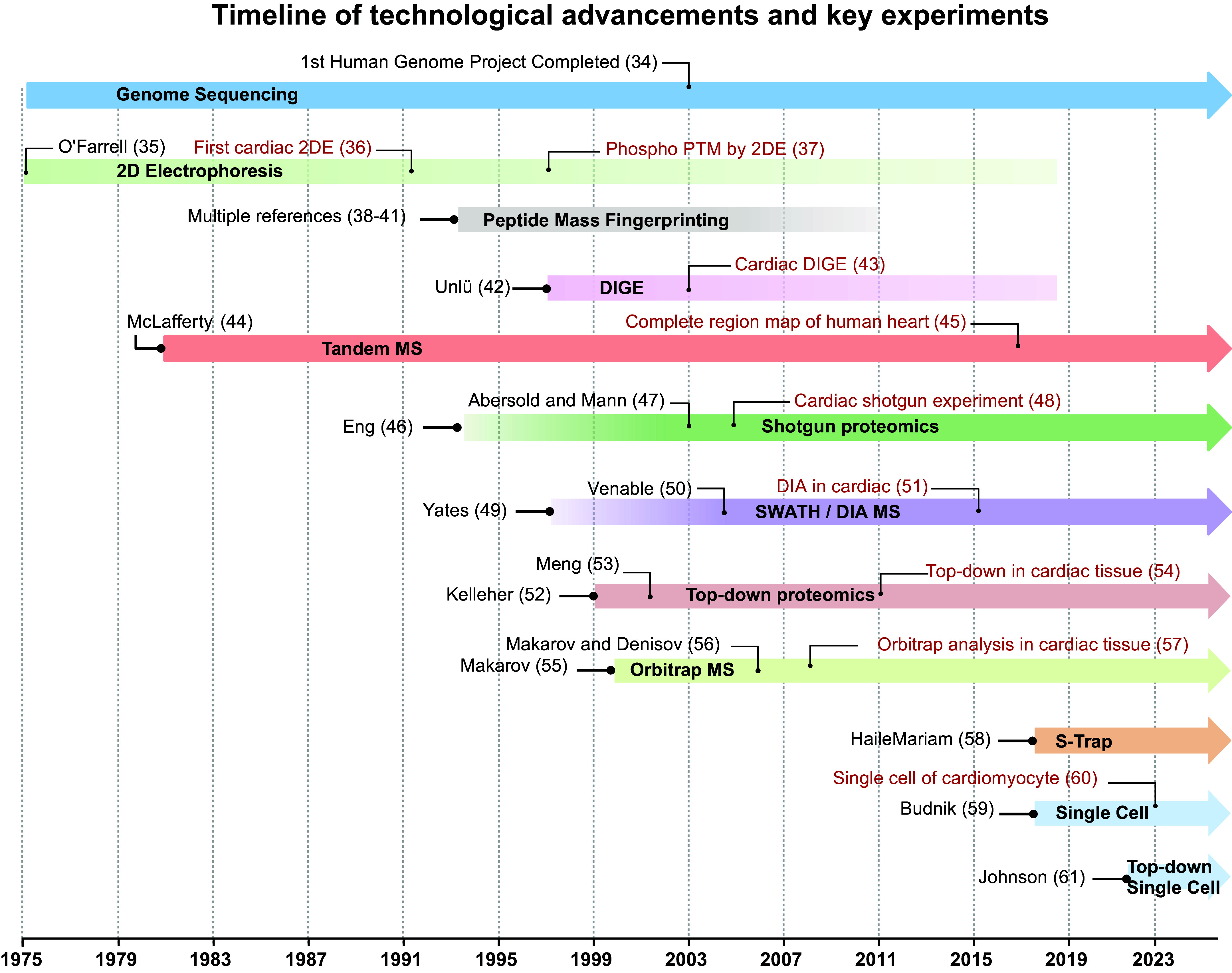
Timeline of proteomic technological advancements and key experiments. Proteomic mass spectrometry (MS) began with separation using 2-dimensional (2-D) gel electrophoresis (2DE) techniques, which was phased out in the coming decades as new approaches were developed. Highlights include the utilization of shotgun proteomics, the invention of the Orbitrap mass spectrometer, and ability to perform data-independent acquisition searches (DIA-MS). Future innovations tend to lean into increased technological fidelity for single-cell resolution proteomics and intact top-down sample acquisition (34–61). DIGE, differential in-gel electrophoresis; PTM, posttranslational modification.

The development of reproducible and quantitative methods for the analysis of the cardiac proteome is an ongoing challenge. Although workflows for cardiac proteomics have been adopted from more general techniques in proteomics, cardiac tissue is inherently challenging to work with. Thus, cardiac proteomics has both benefited from proteomic techniques developed for other cells and tissues and lagged behind other fields. This section provides an overview of the evolution of proteomic characterization and highlights those techniques that were utilized in the early stages of the field to adapt for the challenges of working with cardiac tissue (FIGURE 2).

**FIGURE 3.**
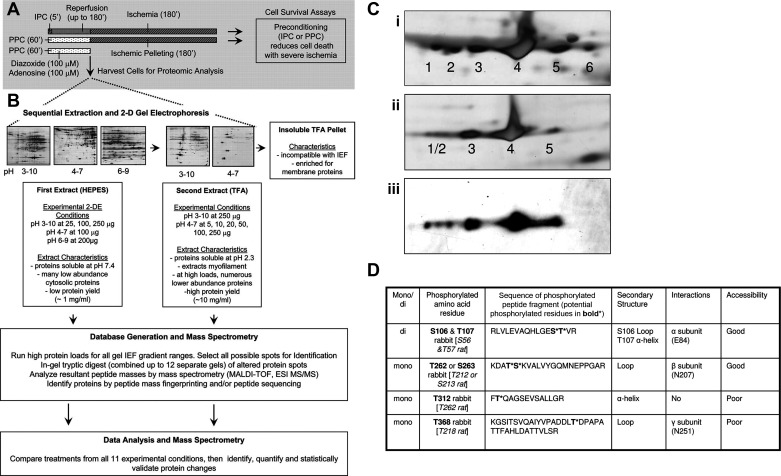
Example of 2-dimensional (2-D) gel electrophoresis (2DE) workflow for rabbit cardiac tissue study on pharmacological preconditioning (PPC; *A* and *B*) and novel finding of ATP synthase β phosphorylation. *A*: example of a timeline of an animal experiment using preconditioning and controls during time of ischemia; numbers with apostrophes represent minutes. IPC, ischemic preconditioning. *B*: sample protocol for a sequential extraction of protein material for a 2-D gel analysis and downstream sample preparation for proteomic mass spectrometry (MS). ESI, electrospray ionization; IEL, isoelectric focusing; MALDI-TOF, matrix-assisted laser desorption ionization-time of flight; MS/MS, tandem MS; TFA, trifluoroacetic acid. *Ci*: silver stain images of the multiple spots of ATP synthase β identified based on liquid chromatography (LC)-MS identification of each spot. *Cii*: silver stain image of dephosphorylated tissue specifically of the region of ATP synthase showing reduction in number of spots was used to ensure that isoelectric point (pI) shift was phosphorylation and not another posttranslational modification (PTM) such as oxidation. *Ciii*: Western blot image of ATPase subunit β confirming identification. *D*: separated 2DE spots from above were cut out and run on a mass spectrometer enriching for phosphopeptides, using immobilized metal affinity chromatography (iMAC) and ESI LC-MS to ensure the unambiguous identification of each phosphorylated residue. Figure adapted from Arrell et al. (298), with permission from *Circulation Research*.

Proteomic characterization of heart tissue has always held great promise, and the opportunity to simultaneously characterize changes in cardiac protein expression during any number of disease states or interventions remains exciting (37, 62–69). Many groups initiated these studies by separating proteins for identification with two-dimensional (2-D) gel electrophoresis (70–76). This technique used isoelectric focusing (IEF) to electrophoretically separate proteins along a pH gradient and spatially resolve them according to their isoelectric point (pI). Isoelectric gradients across a gel were initially hand casted and subsequently replaced by manufactured strips, which provided better data reproducibility (77). Depending on the lysate and protein load, isoelectric focusing could be completed in as little as 3 h or as long as 24 h. Once the first dimension of separation was complete, the strips containing focused protein extracts were loaded onto SDS-PAGE gels, in which proteins were orthogonally separated in a second dimension by molecular weight. The resulting gels were visualized by using Coomassie Blue or a silver staining to yield protein spots, with *x*- and *y*-coordinates corresponding to pI and molecular weight, respectively. Differences between samples were assessed digitally by comparing spot areas or volumes (71). After analysis, protein spots showing differential analysis could be excised, subjected to in-gel digestion, and identified with peptide mass fingerprinting by matrix-assisted laser desorption ionization-time of flight (MALDI-TOF)-based MS analysis or other identification techniques (78–80). Notably, some PTMs added charged moieties to proteins, yielding a pI shift that translated to a spatial difference during isoelectric focusing. A phosphorylation event resulted in a characteristic acidic shift of ∼0.5 pI units, and several early prominent proteins with multiple phosphorylation sites were observed as a train of spots emanating leftward from the unmodified proteoform when viewing the gel images (FIGURE 3) (81–84). Of note, other modifications like oxidation and deamidation, which can be naturally occurring or artificially induced during sample preparation, induce a similar acidic shift in pI, so additional verification is required to make a conclusive PTM determination.

The early excitement surrounding 2-D gel electrophoresis coupled to MALDI-TOF MS was tempered by its technological limitations and the intrinsic challenges of the heart tissue (78). The majority of cardiac tissue by volume is composed of myocytes, and this myocyte dominance can obscure the important contribution of fibroblasts, endothelial cells, and smooth muscle cells (85, 86). Additionally, the requirement for structural integrity of intact cardiac tissue is dependent on strong connective tissues consisting of extracellular matrices (ECMs) rich in collagens, elastins, and fibronectins (87). These highly glycosylated proteins and proteoglycans play a central role in the normally functioning heart, although they are significantly altered in disease, and represent a significant challenge for adequately reproducible tissue disruption and homogenization. At the cellular level, cardiomyocytes exhibit a broad dynamic range, and a predominance of their proteome and volume is composed of contractile myofilament proteins (sarcomeric myosin, actin, tropomyosin, etc.) (88). Though relatively few in number, these proteins of the contractile apparatus occupy a significant amount of the protein by weight in a cardiac tissue lysate. This wide dynamic range that exists between the most and least abundant proteins presented two primary challenges for 2-D electrophoresis methods: First, proteins associated with signaling and maladaptive responses to disease states are generally less abundant, and the sensitivity of protein stains renders visualization of lower-abundance proteins difficult. Second, increasing the protein load to compensate for low sensitivity of less abundant proteins would overload myofilament proteins, whose high abundance and zwitterionic nature would deteriorate the quality of isoelectric focusing in the first dimension. Thus, the attempts to visualize low-abundance proteomes were self-limiting because low-quality gels were not compatible with further analysis or reliable protein IDs (69). The depth of the observable proteome by 2-D electrophoresis was limited to ∼200–300 visualized protein spots, fewer than half of which were identifiable by MALDI-TOF peptide mass fingerprinting (40). Some groups worked to improve IEF conditions for cardiac lysates, accommodating higher loads and expanding the observable proteome by experimenting with zwitterionic detergents for improved solubilization compatible with IEF focusing (66, 89, 90).

Another strategy to address the dynamic range challenge of cardiac tissue involved prefractionating lysate before IEF (91, 92). Although an array of fractionation strategies was developed, their primary goal was to deplete or remove the myofilament proteins and bias the composition of the resulting fractions toward a subproteome of interest. Tissue fractionation typically involves the key steps of homogenization, differential centrifugation, and isolation of subcellular fractions (93). Tissue homogenization traditionally involves a buffer solution that disrupts cell membranes to release the cytoplasmic contents. Homogenates are initially centrifuged at low speed to remove large debris and nuclei, with a subsequent centrifugation at higher speed (maybe including a sucrose gradient) to isolate different subcellular fractions by size and density (94). This fractionation workflow was adapted for cardiac tissues by separately isolating and analyzing the cytosolic, mitochondrial, and myofilament proteomes (95, 96). To that end, the IN-Sequence approach used buffers of different pHs and solubilization strengths to fractionate cardiac lysates, and this technique greatly increased the observable proteome of 2-D gel electrophoresis (93). However, the increase in observable proteome came with a significant increase in work, requiring many more 2-D gels to be run to complete a characterization, and introduced more variability to the processes.

Quantitation was also a challenge. To identify a difference in protein abundance it was necessary to scan a set of stained 2-D gels and measure the differences in mean spot density across conditions. As the observable proteome became larger, more care was required to ensure that the gel spots were correctly matched across a set of experimental gels. Several dedicated software packages were available that could match gel spots across multiple gels (97–99). Aligning 300–1,000 spots across multiple gels was a difficult and time-consuming process, even when software assisted. It also required high-quality separation in each of the gels, which led to considerable optimization and rerunning to get a set of gels sufficient in quality for analysis. A further complication was the consistency of staining. Silver stain techniques were the most sensitive for protein detection but required a user to stop the development of the signal by quenching the reaction (100). This led to variability in staining across gels, even when gels to be compared were developed in the same container. To further complicate matters, silver staining was prone to saturation. Differences in spot intensity could be lost because of overstaining, leading to false negatives. The large difference in protein abundance in cardiac tissue made developing a silver-stained gel particularly challenging, since keeping the highly abundant protein spots from saturating meant sacrificing detection of the lower-abundance spots. Often multiple sets of gels with different amounts of developing would be required to quantify all the observable cardiac proteins.

In the late 1990s a fluorescent labeling technique was introduced by Unlü et al. (42) with the intent of addressing some of the analysis challenges of 2-D gels (101–103). Differential in-gel electrophoresis (DIGE) was a technique in which independent samples were labeled with different fluorescent tags [usually Cy2 (green) and Cy5 (red)]. These labeled samples were combined before first-dimension separation (43). Gel spots with expression differences could readily be observable by the differential labeling and fluorescent tags, which simplified gel alignment and quantitation and provided an expanded range of detection. Once protein spots of interest were identified, gel plugs could be excised and subjected to in-gel digestion, followed by identification via MS (104). The improvements that DIGE brought to 2-D gels shifted the focus of proteomic characterization from the gel separation to MS identification.

The sequencing of the first mouse and human genomes, along with the development of MALDI-TOF MS, represented major milestones in our ability to identify proteins by peptide mass fingerprinting (71, 105). Purified proteins or excised gel spots that were digested with trypsin and analyzed by a MALDI-TOF yielded a characteristic mass spectrum. When these experimentally derived spectra were compared against theoretical spectra whose tryptic sequences were translated from the open reading frames of the newly sequenced genomes, it became possible to assign a confidence value to each spectrum and determine its strongest match. The strength of these matches provided identities to the proteins of interest. With further innovations in separation sciences, 2-D gel-based separations were replaced by chromatographic approaches that could be directly coupled to mass spectrometers. Moreover, with the increase in sensitivity that the MS provided, it became clear that each protein spot on a 2-D gel could actually contain numerous proteins, thus making identification of the protein responsible for the spot change challenging. The development of tandem MS (MS/MS) instruments allowed for individual peptides from a mixture to be selected and isolated for secondary fragmentation, allowing their sequence to be determined directly from a database (106–109). This advancement led to shotgun or bottom-up proteomics, where lysates are digested and separated chromatographically, followed by quantitative MS/MS characterization. This all but eliminated the need for 2-D gel electrophoresis and remains the standard approach for proteomic analyses.

## 4. CURRENT PROTEOMIC TECHNOLOGIES

MS-based proteomic analyses are broadly grouped into three dominant approaches: bottom-up peptide-centric, top-down protein-centric, and targeted peptide/protein acquisitions. In bottom-up proteomics, a protein sample is digested with enzymes such as trypsin, LysC, or GluC and the resulting peptides are chromatographically separated and analyzed by MS (110, 111). These enzymes cleave the peptide bonds at specific amino acid residues to yield predictable peptides. The resulting sample may be more complex in terms of analyte numbers, but peptides are more uniformly sized than proteins, and they lack much of the secondary structure of proteins. This physicochemical homogeneity translates well to chromatographic separations and ionization to yield simplified, quantifiable mass spectra. Once acquired, proteotypic peptides are used to infer the identity and quantity of the proteins from which they were cleaved (110).

By contrast, top-down or “native” proteomics pertains to the direct acquisition of whole, intact proteins by MS (112). This technique can be a powerful tool for the detection and characterization of the array of PTMs on a protein and can provide stoichiometric information with respect to distinct PTM moieties. Identifying large numbers of PTMs with top-down approaches can be challenging compared with bottom-up methods, since enrichment techniques for PTMs often require peptides, and the resulting MS spectra are more complex (113). The choice of bottom-up or top-down (or even the compromise of middle-down where proteins are digested into large peptides) ultimately depends on the research question and on the properties of the protein sample. Ge and colleagues (114) have been leaders in top-down proteomics with respect to heart and heart disease. Her group showed that cardiac troponin I (TnI) phosphorylation was greater in wild-type porcine left ventricle (LV) than in the right ventricle (RV) or the atria but, surprisingly, did not find any significant transmural differences in alpha-tropomyosin, myosin light chain (MLC)-2, or cardiac troponin T (114). This suggests some degree of cardiomyocyte heterogeneity across the heart. Although this initial study focused on only a few proteins, Dr. Ge’s group has carried on to successfully observe extensive proteoform changes to numerous cardiac and skeletal sarcomeric proteins with protein-centric MS (e.g., Refs. 115–125). Excitingly, in the LV of hypertrophic cardiomyopathy (HCM) patients, Tucholski et al. (125) found a complex landscape of proteoforms of sarcomeric proteins arising from combinatorial PTMs, alternative splicing, and genetic variation. This landmark paper, as many of Ge’s laboratory’s papers, sets the stage for the wide-scale implementation of PTMs of all sorts in the heart and in heart disease.

In both cases in which an MS instrument is used to determine the amino acid sequence of the peptide/protein, the sample is subject to analytical separation in line with MS acquisition. This separation is most commonly performed with liquid chromatography (LC), in which peptides (or proteins) are separated by hydrophobicity, or with capillary electrophoresis (CE), in which case the separation is based on electrophoretic mobility. After their analytical separation, peptides/proteins are ionized and introduced into the MS, where their intact mass-to-charge ratio (*m*/*z*) is commonly measured (126). Distinct intact peptides often have identical masses despite having different amino acid sequences. For this reason, proteomics is performed with tandem MS (MS/MS), in which parent peptide ions are fragmented into daughter ions that are subsequently analyzed. Thus, tandem MS generates a spectrum of peaks that corresponds to the *m*/*z* of both intact peptides or proteins as well as *m*/*z* values of fragmented daughter ions to reveal amino acid sequence information. Once acquired, aggregate data are compared with the theoretical masses in a database of all potential fragments that can be generated from known proteins/peptides in a computational workflow that reveals protein- and peptide-level information. After peptide assignment, if two proteins do not have peptide evidence to discriminate them, they will be collected together as a protein group (meaning that the peptide could belong to any member of the protein group). Proteotypic peptides are composed of an amino acid sequence that is uniquely assigned to a single protein, providing unambiguous identification (127). Note that originally proteotypic peptides were defined here as those peptides in a protein sequence that are most likely to be confidently observed by the existing MS-based proteomics methods (e.g., Refs. 128, 129), but the term is now used for peptides that are unique to a specific protein. Of course, the presence of PTMs alters the mass of the amino acid residue to which they are bound, and these altered *m*/*z* can be accounted for as modified and unmodified versions of all proteins in searches within a database (130). Scoring methods have been developed that use the MS/MS spectra to determine the probability of site localization, which is key when there are two or more sequential modified residues. A classic example is phosphorylation of human cardiac troponin I at residues 22 and 23, where the site assignment is based on cleavage of the initiating NH_2_-terminal methionine that results in the mature protein (131, 132).

**FIGURE 4.**
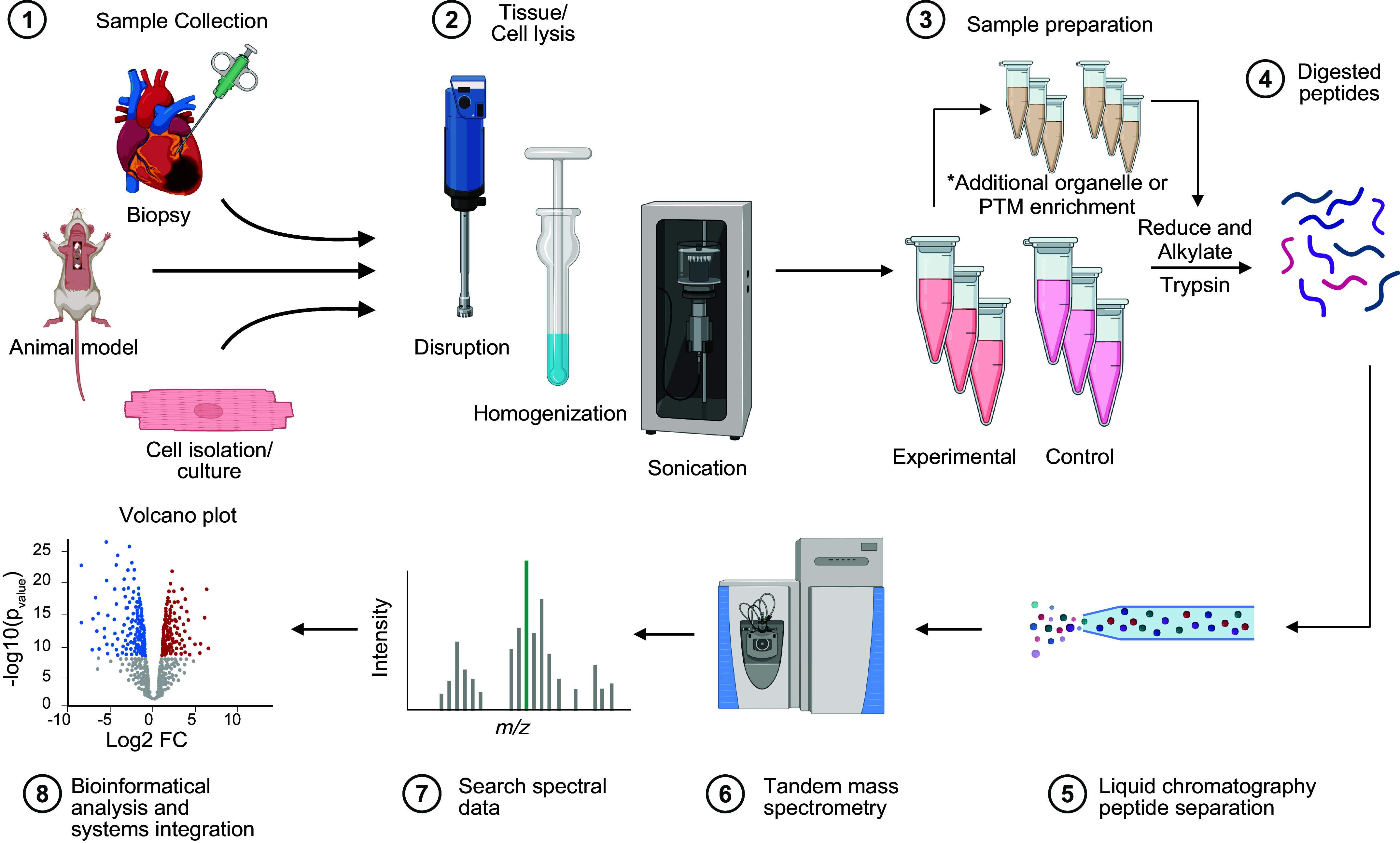
General experimental workflow for cardiac proteomics sample preparation. Tissues or cells are harvested from in vitro or in vivo experiments following experimental procedures. Depending on experiment type, cardiomyocytes may be isolated from complex tissue samples. Cardiac cells and tissues are often lysed by tissue disruption, homogenization, and/or sonication. After lysis, subproteome purification steps such as organelle or posttranslational modification (PTM) enrichment may be undertaken. The protein samples are then reduced to break structural disulfide bonds and alkylated with iodoacetamide or another alkylating agent. Unfolded polypeptides are then digested with trypsin or another tryptic enzyme, creating peptides. These peptides are desalted before being separated by mass on the liquid chromatography (LC) system and ionized as the peptides enter the mass spectrometer. Data are acquired as mass-to-charge ratios (*m*/*z*), which are then deconvoluted in silico, providing a set of peptide concentrations relating to proteins present in the sample ready for downstream statistical processing and analysis. FC, fold change.

Each step in a proteomic workflow, broadly starting with sample preparation, moving to analytical acquisition, and finishing with data analysis and bioinformatic interpretation, has undergone monumental changes over the last 25 years (FIGURE 4). Today, innovations remain focused on increasing the throughput and reproducibility of sample preparation and acquisition, increasing proteomic depth and coverage, improving quantitative accuracy and precision, simplifying workflows, and developing new methods to characterize the functional aspects of proteins. The main technological advances of the last 5 years are highlighted below.

### 4.1. Sample Preparation

Bottom-up proteomic approaches optimally require maximal protein denaturation so that all available cleavage sites are made accessible to proteases to yield a full complement of peptides. This differs from top-down proteomics, where solubilizing by nondenaturing conditions is sufficient. Although several strong detergents such as sodium dodecyl sulfate (SDS) cause effective disruption of van der Waals interactions that maintain hydrophobic domains and underpin many hierarchical protein structures, these can severely suppress the ionization of peptides (133). Their use requires extensive cleanup, and they have been generally considered incompatible denaturants for bottom-up sample solubilization. Several commercially available MS-friendly detergents have been developed to address this challenge (reviewed in Ref. 134). These MS-friendly denaturing agents, which include RapiGest (Waters), ProteaseMAX (Promega), PPS Silent Surfactant (Expedeon), and more recently MaSDeS (135), can both increase the peptide yield of a given protein and enhance the efficiency of membrane protein extraction. In addition to detergent-like denaturants, acidic solvent conditions can denature proteins by disrupting ionic interactions between reciprocally charged amino acid residues (136), though pH must be adjusted to accommodate proteolysis.

More recent preparative strategies immobilize denatured proteins and peptides within a stationary phase. These strategies enable high-efficiency, detergent-based denaturation and cleavage of proteins. Residual MS-incompatible denaturants such as SDS can be removed with aqueous wash steps, while captured peptides are subsequently eluted into a separate receptacle with an organic solvent. This strategy lies at the heart of several commercially available formats including filter-aided sample preparation (137), S-trap (138), on-pellet digestion (139–141), SP3 (142), and SP4 (143). These trapping methods have been adopted to address several other more specialized proteomic research questions such as the selective enrichment of cysteine residues (144).

In addition to the growing preparative repertoire, the choice of available proteolytic enzymes has also expanded. Owing to its robust characterization, efficiency, solvent flexibility, and peptide yield, trypsin remains the predominant protease for bottom-up proteomics. Other digestion techniques being developed take advantage of the unique properties of alternative proteolytic enzymes to address specific research questions. Histone proteins, for instance, are rich in the arginine and lysine residues that lie adjacent to trypsin cleavage sites; tryptic digestion of such proteins yields peptides that are neither sufficient in numbers, sufficiently proteotypic, nor adequate for MS acquisition. By contrast, ProAlanase (also known as An-PEP or EndoPro) is a protease from the fungus *Aspergillus niger* that cleaves proteins at the COOH terminus of proline and alanine residues. These alternative enzymes require altered workflows to suit their optimal biochemical profiles; ProAlanase, as an example, has optimal cleavage activity under acidic conditions at pH 1.5 (145). Similarly, proteolytic enzymes are increasingly being engineered for characteristics to specifically cleave proteins in a way that enables accurate proteoform and PTM analysis (see for review Refs. 146, 147). To that end, a modified form of chymotrypsin (chymotrypsiN), engineered to exclusively cleave a peptide bond adjacent to modified asparagine residues, can be used to reveal buried N-linked glycosylation sites (148).

### 4.2. Automation of Sample Preparation

Automated and semiautomated sample preparation workflows are increasingly adopted by proteomic research laboratories because they can enhance the reproducibility and robustness of experimentally derived data (149). The individual steps involved with proteolytic sample preparation, each with its own inherent variance, ultimately coalesce to yield a quantitative set of peptides. Reliable sample processing as well as accurate troubleshooting and continuous improvement are dependent on minimizing the individual and collective variance of these steps. Given the increasing sensitivity and precision of modern analytical instruments, workflows are increasingly reliant on automated liquid handling workstations to perform pipetting, mixing, and other volumetric sample handling tasks. Diligent assessment of the sample type, protocol design, reagents, and incubation conditions can significantly improve the speed and consistency of preparation.

An early semiautomated in-gel digestion protocol for bottom-up proteomics was reported using a Bravo workstation introduced by Agilent (150). Fully automated protocols were subsequently reported using NPx and i7 automated workstations by Beckman Coulter (150, 151). Automation is now relatively widespread, with a host of systems used for plasma and cell processing. The challenge inherent to cardiac and aortic samples continues to be reliable and reproducible ECM subfractionation techniques that overcome the dominance of the nearly insoluble myofilament contractile proteins. Nevertheless, progress in this arena has involved the combination of semiautomated workflows with tissue extraction instruments such as a Barocycler (152) that utilize pressure and temperature to homogenize challenging tissue types including formalin-fixed and paraffin-embedded aortic samples (153).

### 4.3. Accuracy and Proteome Coverage

Continuing innovations in LC and MS instrumentation have led to improved LC reproducibility, increased sensitivity, and faster MS instrument scan cycles. These improvements have fostered creative alternatives to MS data acquisitions from a complex peptide mixture. A key development in this arena was the advent of data-independent acquisition-MS (DIA-MS). The traditional approach to MS-based proteomics was called data-dependent acquisition-MS (DDA-MS), or “shotgun proteomics,” in which the most intense precursor ions were selected for fragmentation and the resulting files were searched against a fully known species-dependent peptide library representing all known proteins (49). Thus, DDA-MS has a tendency to undersample and/or miss lower-abundance peptides that may coelute with higher-abundance peptides, which can lead to a significant amount of “missing data.”

By contrast, DIA-MS acquires spectra by sequentially scanning defined mass-to-charge (*m*/*z*) windows in a systematic and unbiased manner. This approach yields more complete and consistent measurements across the entirety of the proteome but exhibits a significant increase in the complexity of fragmentation spectra (50, 154). The ensuing data-rich MS files are typically searched against a preestablished library of experimentally observed fragmented peptides that are related to the sample in question, combining both fragmentation and chromatographic features (peptide query parameters, PQPs) (155). DIA-MS identifications are searched by matching observed ion features with the PQPs in the spectral library (contrasted with a peptide mass library search in DDA-MS) (156). More recently, search engines such as DIA-NN, MSFragger, and PECAN can impute in silico spectral libraries to perform library-free analysis on the DIA-MS data, relieving the need to acquire an extensive library presearch (157–160). One still observes missing data, but the inference is that the peptide or protein analyte is below the limit of detection rather than missed because of stochastic sampling. In this way, DIA-MS could be considered analogous to sandwich immunoassays (ELISA), where each protein analyte has a defined lower limit of detection and a lower limit of quantification, which is also the case with targeted MS assays. DIA-MS approaches have been used in a number of cardiac-specific experimental approaches, some of which are outlined in Refs. 161–164.

### 4.4. Protein Quantification

Accurate measurement of the quantity of proteins in a proteomic screen is critical to making differential comparisons between states. There are several strategies to perform MS-based quantitation. Signal intensity can be compared for each detected species between groups of samples with label-free methods or with metabolic labeling and/or postdigestion labeling approaches (22, 165).

In traditional DIA-MS and DDA-MS label-free studies, protein quantification is based on peptide quantification. Chromatographic peak area (MS1) or summation of the top few precursor fragments (MS2) of the peptide represents the signal intensity for each peptide and, ultimately, proteins. Label-free quantitation requires highly reproducible sample handling and analysis protocols. Since each sample is analyzed independently, there is an increased opportunity for error (149). Instrument performance may be prone to drifting across a cohort of samples in smaller sample runs. In the case of larger experiments, samples may be prepped and run in batches separated by time, creating the potential for a batch effect in the quantitation, due to subtle differences either in sample preparation or in instrument performance. Batch effects can be addressed by corrective software discussed below.

Whereas sample preparation techniques are increasingly automated and multiplexed, MS is inherently sequential in nature: samples are injected, separated, and acquired one at a time. In parallel with the evolution of fluorescent labeling of spots on a 2-D gel, MS throughput can be increased with approaches that tag proteomes from individual samples, allowing them to be combined and acquired in tandem and differentiated after acquisition. To that end, tandem mass tag (TMT) and isobaric tag for relative and absolute quantitation (iTRAQ) reagents involve the covalent attachment of a stable isotope-containing mass tag to the NH_2_ terminus and/or to a lysine residue on peptides within a digested sample. TMT or iTRAQ reagents contain a unique set of stable isotopes that can be used to label anywhere from 2 to 8 (iTRAQ) or 18 (TMT) different samples, which then can be combined and analyzed within a single MS acquisition (166). Although the labeled peptides are simultaneously acquired, they are independently quantified based on the unique isotopic reporter ion masses assigned within each sample. Thus, deconvoluting these “tags” provides highly accurate relative quantitation from a single MS run. Some early adopters in the cardiac space deployed TMT labeling as part of a phospho-proteomics study on remote ischemic preconditioning (167), along with the related dynamic intracellular *O*-linked-N-acetylglucosaminylation (O-GlcNAcylation) PTM of myofilament proteins (168). TMT tags have also been used to characterize the impact of expression of the embryonic transcription factor Tbx18 in neonatal rat ventricular myocytes to investigate induction of cardiac pacemaker cells (169). Tbx18 was found to induce wide-ranging reprogramming of the myocytes including increased expression of pacemaker channels and extensive cytoskeletal and extracellular matrix remodeling consistent with invocation of the epithelial-to-mesenchymal transition program. TMT is only one of many isobaric labeling reagents, some of which are more cost effective and have improved reporter ions that minimize the typical dynamic range compression inherent in these multiplexed experiments (170). To that end, deuterium and *N*,*N*-dimethyl leucine residues have also been used as isobaric mass (171, 172).

The final approach to quantitative proteomics is metabolic labeling, usually done by stable isotope labeling with amino acids in cell culture (SILAC) (173, 174). In this approach, proteins are metabolically labeled with isotopically heavy or light versions of lysine or arginine during cellular growth. The samples are then harvested and combined for digestion and subsequent MS analysis, where the heavy- and light-labeled peptides can be resolved and quantified in the MS1 scan. Although this approach was initially carried out during cell culture, the isotopic labeling can also be completed in small mammals, among other organisms. In 2011, Scholten et al. (175) used mouse hearts in tandem with SILAC to perform quantitative proteomics for the characterization of the right and left ventricular proteomes. They identified >3,700 proteins and were able to quantify ∼2,000 of them, indicating a high degree of similarity between the proteomes of the ventricular structures.

Labeling methods improve throughput by enabling the simultaneous acquisition of multiple samples. In addition to these multiplexing strategies, there are also a number of ways to speed up the LC-MS runs themselves. The main parameter through which to achieve faster runs is to speed up the chromatography through steeper chromatographic gradients and/or increased flow rates. Although this seems self-evident, doing so comes at the expense of chromatographic resolution, decreased peak capacity, and detrimental quantitative accuracy due to fewer data points across a peak. At the spectrometer, one can also reduce overall instrument time by decreasing or eliminating the time devoted to sample loading and column equilibration. These strategies that maximize instrument utilization time are increasingly important, especially for large sample cohorts. Adopting a parallelized dual-trap setup with a single column is one strategy that we have found useful (60). Likewise, the Evosep system, in which desalted peptides are loaded onto C18 stage tips and subsequently eluted at low pressure and captured in a storage loop where they await direct and automated loading onto an analytical column, has also increased throughput for large cohorts (176). Faster-scanning MS instruments can similarly acquire data at a higher speed without loss in sensitivity or resolution. In general, these improvements can accommodate faster LC gradients or yield deeper, more comprehensive proteomes from similar LC gradients. The integration of a third, ion mobility-based separation parameter, either as an interface between the LC and the MS or integrated within the MS instrument itself, has endowed MS workflows with additional improvements in resolution. In contrast with reverse-phase chromatography, in which peptides are separated on the basis of their hydrophobicity, and in contrast with the MS itself, which analyzed peptides based on their mass-to-charge ratios, these ion mobility modules serve to separate ionized peptides according to their size, shape, and charge. Several configurations of this separation are routinely used, including high-field asymmetric waveform ion mobility spectrometry (FAIMS) and multiplex ion mobility spectrometry (IMS), which can improve proteome coverage, reducing the need for preinjection fractionation or enrichment. The timsTOF system from Bruker houses two parallel IMS tubes that work in tandem: one accumulates and traps IMS-separated peptides while the other serially releases its trapped peptides for fragmentation and MS/MS analysis, achieving faster sequencing speeds without decreasing sensitivity (177). We have recently used a FAIMS setup to replicate the proteomic coverage previously obtained by three-part “IN Sequence” fractionation of heart tissue, with a single-step sample processing followed by gas-phase separation (178). Although these strategies can expedite MS acquisition, they can require careful optimization and validation to ensure that the results are reliable and reproducible. However, MS instruments continue to evolve, as evident by the recent release of the Orbitrap Astral MS from ThermoFisher including the novel Astral Mass Analyzer, promising great protein coverage with a higher throughput (179–181). Today, the MS instruments are increasingly reliable, and when they are coupled with automated sample preparation the remaining challenges are primarily data analytics and data interpretation, at least for protein quantification.

### 4.5. Targeted MS Approaches

In addition to the primarily discovery-based techniques described above, a growing field in proteomics is the use of targeted MS approaches. In these approaches a prescribed set of proteins or proteoforms is specifically measured instead of taking an unbiased survey of the proteome. Targeted assays typically have lower coefficients of variance (CVs) and have a shorter run time than discovery approaches. This can be achieved by using the MS-based multiple reaction monitoring (MRM) technique. MRM uses a triple quadrupole MS to only target ions corresponding to a peptide of interest for fragmentation, producing a range of daughter ions. Only ion species that match the specific parent and daughter masses (also known as transitions) will be used for quantitation. By ignoring all other ions, this technique can achieve high sensitivity, precision, and accuracy (182). A further advantage of MRM assay is the use of isotopically heavy labeled (^15^N or ^13^C) versions of target peptides that can be added to a sample to obtain an absolute quantitation of an analyte. Individual MRM assays can be combined into a multiplexed assay to measure a series of analytes in a single run. This technique was used in the analysis of cardiac Troponin I (TnI) phosphopeptides in failing myocardium. MRM assays were established for 14 sites and their unphosphorylated versions in TnI. Analysis of human explanted failing hearts compared with nonfailing donor hearts revealed modulation of TnI phosphorylation pattern with disease progression (183, 184). These findings were further confirmed in a canine model of HF and reversed with resynchronization therapy (183). A similar approach was also used to characterize phosphorylation sites in the cardiac mitochondria (185). Expanding the MRM-based toolkit, a multiplex protein assay has also been developed in our laboratory to observe mitochondrial function. The MitoPlex assay targets 37 proteins critical to central carbon chain metabolism and overall mitochondrial viability (186). This assay has been used in conjunction with related metabolite analysis to quantify mitochondrial function.

### 4.6. Bioinformatics and Data Analysis

**FIGURE 5.**
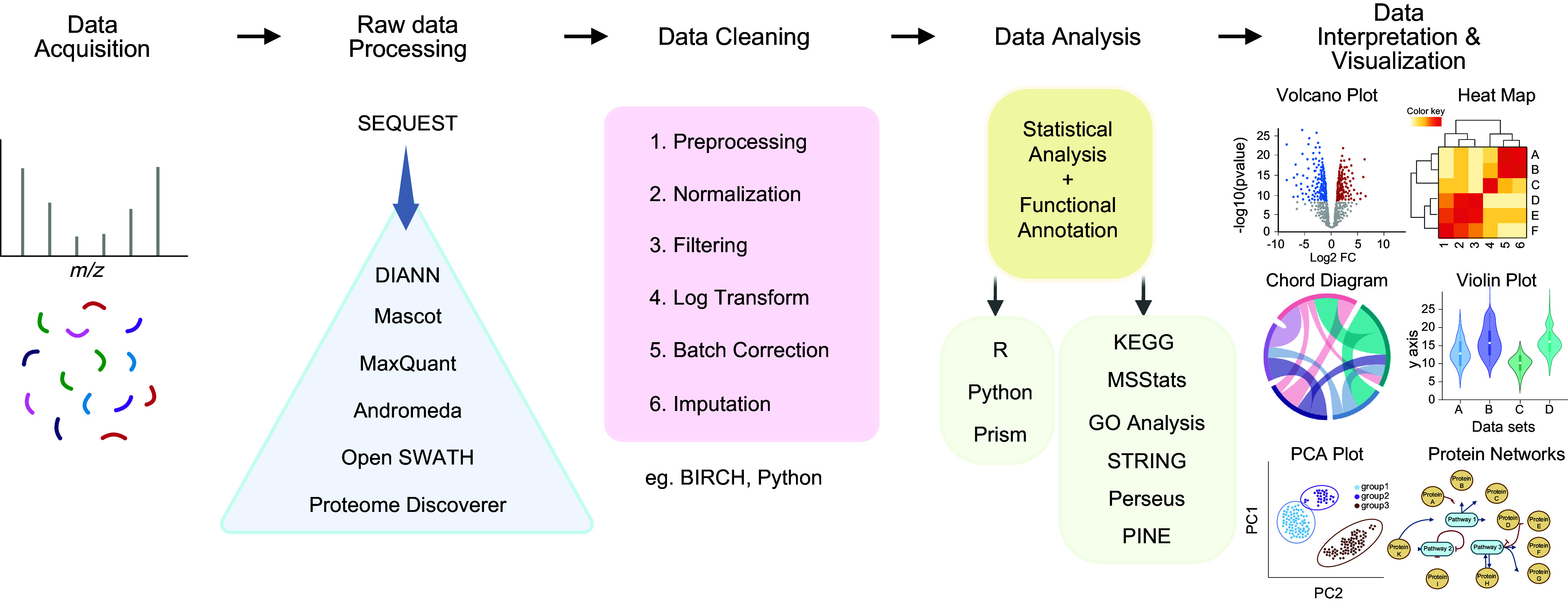
Overview of common data analysis workflows and tools for proteomic data. Raw data files are acquired from the mass spectrometer as spectra comprising of mass-to-charge ratios (*m*/*z*). The peaks in the data are then assigned to masses and processed with a variety of software packages. Optional library generation can take place, depending on whether the platform and workflows are data-dependent acquisition (DDA) or data-independent acquisition (DIA) based. Large data arrays then undergo further statistical processing, filtering, and corrections to have normalized and comparable results. Finally, data are analyzed and may be visualized with a number of software tools to gain biological insights about the completed experiment. FC, fold change; PCA, principal component analysis.

Bioinformatic tools are essential for the analysis and interpretation of proteomic data. These tools use various algorithms to identify and quantify proteins, assign biological significance to the identified proteins, visualize the resulting data, and enable researchers to extract meaningful information from large and complex datasets. An overview of the current workflows is presented in FIGURE 5.

There are numerous search engines for peptide identification, which match and assign MS spectral data within a sequence database to identify the peptides in the sample. The identified peptides are then used to infer the identity of the proteins from which they are derived. Popular search engines include Sequest, which was originally developed by Jimmy Eng and John Yates III (46, 187), which alongside Mascot (188) paved the way for an array of novel search algorithms like MaxQuant (189), Andromeda (190), the software package Proteome Discoverer (191), OpenSWATH (192), and most recently DIA-NN (157). The aforementioned search engines each employ scoring functions based on the number of matched peptides, quality of the mass spectra, and false discovery rates (FDRs) of mass identification. After mass identification, peptides that consist of an amino acid sequence that is unique to a specific protein are assigned to that protein, while peptides whose sequence is shared among several proteins are assigned to a protein family. Protein quantification tools use various algorithms to determine the abundance of the identified proteins with quantitative processes such as spectral counting, label-free quantification (LFQ), isobaric tagging, and statistical analyses to identify differentially expressed proteins. The advent of machine learning-based tools has also led to the increasing adoption of this technology in proteomic analysis. For example, Random Forest and support vector machines (SVMs) have been used for protein classification, peptide identification, and prediction of PTMs (193). These tools use various algorithms to identify patterns in datasets, allowing researchers to make predictions based on the learned patterns. When hundreds of samples are analyzed, additional batch corrections may be required for sufficient data QC. One software tool capable of providing this type of correction is Birch, which pairs an easy-to-use GUI platform with robust capabilities for proteomic sample batch correction (194), although other platforms are also available (195–200).

Functional annotation tools that assign biological significance to the identified proteins are most often based on gene assignment, which are less simple as one might expect. Gene Ontology (GO) analysis and pathway analysis tools such as Ingenuity Pathway Analysis (IPA) (201) and KEGG (202) are some of the more popular functional annotation tools available. DAVID (203) and GO (204) analysis use statistical methods to assign proteins to functional categories based on their biological processes, molecular functions, and cellular components. Pathway analysis tools use curated databases to assign proteins to pathways, allowing researchers to understand the broader biological context of identified proteins. Data visualization tools help researchers in identifying patterns and relationships between proteins. Heatmaps, principal component analysis (PCA) plots, and volcano plots are some of the more ubiquitous data visualization tools used in proteomic analysis. Heatmaps allow researchers to visualize the expression patterns of proteins across samples, whereas PCA and volcano plots allow researchers to identify relationships between proteins and to identify differentially expressed proteins between samples. Our own group has developed a platform called PINE, which allows easy manipulation of discovery-based proteomics data coupled with downstream visualization for use with these types of databases (205), whereas MacCoss and colleagues have an alternative tool for targeted MS proteomic data with Skyline (206) and Panorama (207).

## 5. PROTEOMES OF THE HEART AND ASSOCIATED PATHOLOGIES

Characterization of the cardiac proteome can be challenging. The physical characteristics and access to tissue samples can limit the depth and power of an analysis. As discussed above, cardiac tissue is composed of many cell types, specialized regions, extensive extracellular matrix, and, within the myocytes, an abundance of the contractile myofilament proteins associated with the organ’s primary function. Selection of a sample type and preparation for proteomic analysis depend entirely on the goals of the experiment. Sample types can range from biopsies obtained from a clinical cohort to tissues harvested from animal models to manipulation of isolated primary or cultured cardiac cell types. Once obtained, for traditional bottom-up proteomics samples can be maximally solubilized for digestion or processed in a manner to preserve and enrich a subproteomic characteristic, like an organelle or PTM population. The principles of tissue and cell homogenization are well reviewed in Refs. 208, 209, and subproteomic fractionation options are discussed in sects. 6.1 and 6.2.

One of the most challenging aspects of cardiac proteomic investigations is the collection of human heart tissue samples. These studies require committed clinical collaborators to obtain biopsy or explanted tissues from an identified patient population as well as, and even more challenging, from nonfailing or healthy donors. Sample sizes are often limited, and interpretation is further complicated by the comorbidities of the patients. In a landmark study by Doll et al. (45), the authors characterized 16 distinct regions, surveying the major anatomical structures and measuring three cultured cell types from healthy human hearts. By dividing each digested sample over eight fractions by reverse-phase chromatography they identified >11,000 proteins across the dataset. Among several insights, the authors reported the percentage of each organelle in cellular protein mass across heart regions, demonstrating the relative abundance of myofilament and mitochondria particularly in the contractile tissues of the atria, ventricles, and septa. The detailed analysis allowed the authors to use their dataset to compare with biopsy samples from atrial fibrillation patients. Although this is only a proof-of-principle investigation, they have found several interesting differences in the mitochondria of atrial fibrillation patients’ left atrium compared with control.

Considering the challenges of collecting human cardiac samples and the inherent heterogeneity of the cellular populations, animal and cell-based models are an invaluable resource for proteomic characterization. Akin to the Doll et al. study in humans, there have been several efforts to characterize the cardiac proteome in other systems. In 2006, Kislinger and colleagues (210) presented a full proteomic characterization of six different mouse organs, including the heart. Their approach was to divide each tissue into subcellular fractions by ultracentrifugation (cytosol, membrane, mitochondria, and nucleus). This same group reprised their analysis with another more detailed characterization of the mouse heart proteome (211). In this study, the authors analyzed more subcellular fractions to identify almost 5,000 proteins. Similar database-style characterizations have also been carried out in nonhuman primates (212). The most comprehensive of these studies has been by Linscheid et al., who did a multispecies comparison of heart tissue from the left and right atria and ventricles. They identified >7,000 proteins in human, pig, horse, rat, mouse, and zebrafish hearts and compiled an online comparative database (213, 214). This work creates an essential link when inferring animal model data to humans.

There is also significant value in deconvoluting the multiple cell types that make up cardiac tissue. The heart is a complex organ, and characterizing the composition of the individual components is critical to understanding the integration of those parts in the whole. Isolating individual cell types for proteomic analysis provides the opportunity to view their individual molecular composition. Proteomic analysis of isolated cell types has been performed by many groups (215–217). A recent example was performed by Poulsen et al. (218), where the authors characterized sorted cardiomyocytes and fibroblasts from rat hearts. Using a prefractionation approach they have identified over 5,200 and 6,300 proteins from the cardiomyocytes and fibroblast cells, respectively. These findings provide an excellent benchmark for comparing expression profiles with transcriptomics, between cell types and comparing with models of disease. This type of analysis has recently been further extended to include techniques for the isolation of cardiomyocytes from cryopreserved human cardiac tissue followed by flow cytometry (88, 219).

### 5.1. Heart Failure and Its Proteomic Alterations

Proteomics has played a significant role in advancing our understanding of heart failure (HF) by providing insights into the molecular mechanisms and changes in protein expression associated with the disease (64, 65, 220, 221). HF is a complex clinical syndrome that stems from a wide range of underlying pathophysiological mechanisms that lead to impaired cardiac output that falls below the demands of the body because of impaired systolic and or diastolic function (222, 223). Patients suffering from HF can present with reduced (HFrEF) or preserved (HFpEF) ejection fraction (155). HFrEF is predominant in males and is usually associated with loss of myocardium (224). HFpEF is a heterogeneous clinical syndrome most common in women in which LV ejection fraction (LVEF) is maintained to at least 50% of normal and is associated with several noncardiac comorbidities (225–228).

Proteomic analyses have provided insights into HF in several ways, including identifying potential biomarkers for early detection and diagnosis, understanding the underlying molecular mechanisms of the disease, and characterizing potential therapeutic targets. The fundamental approach to proteomic research on HF involves comparing the proteins derived from healthy hearts with those derived from the failing heart. This experimental approach has been performed with techniques ranging from 2-D gel electrophoresis to DIA-MS using cells, animal models, and human tissues. By comparing these proteomes, researchers have identified proteins that are differentially expressed in compromised or failing hearts, revealing new mechanistic insights into the development and progression of this multifaceted disease. To that end, proteomic analysis has been instrumental in characterizing and understanding HF (see for more detailed reviews Refs. 229–234). In sects. 5.2 and 5.3 we highlight several recent studies utilizing patient samples or animal models to understand this complex disease.

### 5.2. Human Studies of HF and Myocardium Remodeling

In an innovative study, Linscheid et al. (235) collected heart biopsy samples from patients during valve replacement surgery. They obtained biopsies from the right and left atria and left ventricle of seven male patients with mitral valve regurgitation, dilated left atria, but no history of fibrillation. Proteomic analysis identified >7,000 proteins across the samples and shed new light into the myocardium’s response to left atrial dilation and conditions for the development of arrhythmogenesis. Brandenburg et al. (236) used a proteomic approach to interrogate the various hemodynamic subtypes associated with arterial valve stenosis. In this pathology, a large range in left ventricular function can occur. DIA-MS was utilized to evaluate proteomes of endomyocardial biopsies from four different subclasses of patients in their study, spanning the clinical presentations of arterial valve stenosis. Among their findings, a dysregulation of the ryanodine receptor was observed in the patient groups exhibiting reduced left ventricular function.

To understand the mechanisms of advanced HF, Liu et al. (237) combined a quantitative labeling approach with LC-MS/MS analysis to characterize proteomes from LV tissue of end-stage patients suffering with dilated cardiomyopathy arising from a previous myocardial infarction (MI). By comparing these tissues with healthy control subjects, they identified 125 differentially expressed proteins involved in membrane organization, mitochondrial organization, translation, protein transport, and apoptosis (237). Another multiomics approach characterizing chronic HF tissues from ischemic and nonischemic patients yielded similar findings and identified that potentially convergent regulation occurs to the processes underlying advanced HF (238). The timing of when this pathophysiology occurs is unclear, thereby impacting the time that therapies could be applicable. Furthermore, a recent study used a DIA-MS approach to investigate proteomic signatures in the myocardium of HFpEF patients. LV biopsies were compared with a mixed control group to show alteration in a wide variety of pathways, including mitochondrial dysfunction, oxidative stress, and inflammation (239).

An important study by Previs et al. (240) utilized a multiomics approach in tissue from failing and control hearts to understand how the altered energetics that develop during hypertrophic cardiomyopathy (HCM) could be the cause of affected contractile function. Their analysis revealed a significant reorientation of energetic pathways in HCM hearts, culminating in decreased ATP, among other cofactors, and reciprocal increases in ketone bodies and branched-chain amino acids. Subsequent functional analysis demonstrated that decreased ATP consistently aligns with impaired myofilament cross-bridge attachment.

Chronic heart disease impacts the mitochondria with extensive rewiring of the proteome that includes altered gene translation and redox regulation as the heart tumbles into dysfunction. No one process, however, has been identified to be the dominant initiating factor that drives HF. One plausible possibility is the dysregulation of the kinase/phosphatase signal transduction arrays. In a comprehensive study, Reitz et al. (241) performed a proteomic and phosphoproteomic analysis of explanted LV tissue from dilated cardiomyopathy (DCM) and ischemic cardiomyopathy (ICM) patients compared with control subjects. The authors identified >5,000 proteins and 13,000 phosphosites across their analysis and found many region- and etiology-specific signaling pathways outside of altered mitochondrial function. This included a specific hyperphosphorylation of an intercalated disk protein, αT-catenin, particularly within DCM patients compared with control subjects. αT-catenin is a testis-specific, developmentally dispensable protein isoform, which binds to actin filaments and is involved in cell-cell communication (242). This proteomic analysis uncovered a novel phosphorylation cluster consisting of S637, S647, T649, and S650 that were exclusively found in tissues from DCM patients. Interestingly, S650 is a site for a potential single-nucleotide polymorphism variant that is linked to arrythmias (241, 242).

### 5.3. Animal Models of HF and Myocardial Remodeling

An essential component of understanding the dynamic proteome during the development of HF has been the use of animal models. The various animal model phenotypes of human HF have been extensively reviewed elsewhere (243, 244). One of the more common models employed for HFrEF is permanent coronary artery ligation (PCAL), which results in myocardial ischemia and subsequently MI. This model induces HFrEF as a function of the loss of viable myocardia resulting in left ventricular remodeling. As an example, Bai and colleagues (245) performed quantitative proteomic and metabolomic analyses on heart tissues derived from PCAL rats to reveal alterations in many key cardiac signaling pathways after MI. These pathways notably included metabolism as expected (e.g., amino acid metabolism) but also proteins involved in vascular smooth muscle contractility, formation of gap junctions, and neuroactive ligand-receptor interaction (245). This same approach was applied in a post-MI mouse model to reveal acute changes in MI tissue, including a marked increase in vitamin D binding protein (246). Within the context of larger-animal models, proteomic analyses of sarcomeric myofilament proteins derived from a swine model with PCAL revealed alterations in cardiac troponin T and cardiac myosin heavy chain proteins (247). However, one of the most telling studies was in the PCAL model investigating the response to immediate administration of a potential postinfarction therapy, specifically glucagon-like peptide-1 receptor (GLP1R) agonist. Proteomic analysis was performed on left ventricular tissue during a time course to evaluate differences in maladaptive remodeling compared with vehicle-treated PCAL mice and demonstrated mitigation of post-MI remodeling with GLP1R agonist administration (248). The proteomics clearly illustrated an alteration to several mitochondrial pathways including respiration, oxidative phosphorylation, as well as glycolysis and fatty acid beta-oxidation, suggesting a potential acute mechanism for the drug agonist within the heart that may not be directly related to its glucose-dependent stimulation of insulin secretion.

The spontaneously hypertensive HF (SHHF) rat and the Dahl salt-sensitive (DSS) rat represent two commonly used genetic models of HFpEF. SHHF rats develop diastolic dysfunction and LV hypertrophy as they age, which ultimately progresses to HFpEF. Proteomic analyses of myocardium obtained from SHHF rats have been performed with 2-D gel electrophoresis techniques to observe changes in energy metabolism and mitochondrial function (249–251). In contrast to the spontaneous development of HF seen in the SHHF rat, hypertension and LV hypertrophy can be induced in the DSS rat model with a high salt intake. Beyond hypertension and hypertrophy, DSS rats also exhibit diastolic dysfunction and fibrosis that resembles some of the human HFpEF phenotype. Several proteomic studies of DSS rats have found altered inflammatory responses, mitochondrial fission, as well as glycoproteomic changes (252–254). The DSS rat model has also served as a platform for pharmacological studies, and how this would translate to human HFpEF is not yet clear. To that end, the administration of canagliflozin, a sodium-glucose cotransporter 2 (SGLT2) inhibitor that lowers blood sugar by increasing glucose in the urine, in a DSS rat model resulted in improved outcomes pertaining to myocardial hypertrophy, fibrosis, and left ventricular diastolic dysfunction. Based on the proteomics, the myocardial metabolism was associated with an upregulation of proteins involved with AMP-activated protein kinase (AMPK), sirtuin 1 (SIRT1), and peroxisome proliferator-activated receptor gamma coactivator-1 alpha (PGC1α) pathways (255). The use of SGLT2 inhibitors has been shown to benefit patients with HF including HFpEF, with or without type 2 diabetes (256, 257). With remote conditioning, there is also the concept of secreted players that provide a signal from one organ to another (preconditioning is discussed in sect. 5.5) such as the signals traveling from kidneys to the heart (258). Proteomic analysis of secreted proteins under endoplasmic reticulum (ER) stress has led to unique signaling molecules that previously were unknown (259). Proteomics on secreted proteins as well as exosomes [extracellular vesicles (EVs)] have also been carried out in context to identify potential biomarkers or for their application as therapeutics (260, 261). EVs are small membrane-bound vesicles that are released by cells to play a role in paracrine and autocrine communication (217). Proteomics-based methods for their isolation from specific cells or from biofluids continue to grow (e.g., Refs. 179, 262, 263). Marbán’s group has paved the way in the area of EV therapeutics (264, 265), EV proteomics (266, 267), as well as their consequences in the potential remodeling in the heart. This included extensive phosphorylation of the myofilament proteins, which in turn correlated to functional recovery of the DSS HFpEF animal heart that could portray involvement of the PKC isoform through increases in PKCα, β, and δ concentration with HFpEF, whereas EV treatment led to a reversion of PKCβ action and its proteomic signature (268).

Proteomic analyses have similarly been utilized to investigate how the RV and LV myocardial tissue differ in the development of hypertrophy. By constricting either the descending thoracic aorta or the pulmonary artery of rabbits, Friehs and colleagues (269) were able to selectively increase the pressure in the RV and LV. A quantitative shotgun proteomics approach uncovered divergent protein expression profiles between the left and right hypertrophy, revealing differences in oxidative phosphorylation, structural proteins, and calcium handling (269). An excellent review published in 2021 compiled the studies using proteome determination to better appreciate the molecular changes occurring during the cardiac myocardial remodeling (270). An LC-MS approach was applied to the characterization of proteome changes involved in DCM induced by Coxsackievirus B3 (CVB3) infection and taking place after MI, compared with control mice (271). It was demonstrated that regulations varied between the two mechanisms that can cause HF via LV hypertrophic remodeling. Broad biological processes were altered, including biosynthetic, metabolic, membrane organization, protein folding, and were upregulated in ICM hearts compared with DCM hearts. In addition, centrosome localization, electron transport chain, and several other biosynthetic and metabolic processes were downregulated (271).

### 5.4. Reversible Ischemia and Associated Pathophysiology

Myocardial ischemia-reperfusion injury (IRI) is a complex cascade of cellular injury that paradoxically occurs when blood flow is restored to an occluded and ischemic region of the heart. IRI results in significant damage to the myocardium and can result in various forms of heart disease ranging from cardiac arrhythmias to heart failure and MI. The pathophysiology of IRI involves a complex interplay of cellular and molecular events that involve uncoupling of mitochondrial electron transport, inflammation, apoptosis, and necrosis (272–276). The process of myocardial IRI involves two distinct phases: The ischemic phase is characterized by impaired blood flow to the heart resulting in an insufficient oxygen and nutrient availability that disrupts mitochondrial ATP turnover, increases intracellular calcium levels, and induces acidosis in myocytes. In the reperfusion phase, although restoration of blood flow to the heart is inherently critical, the altered and uncoupled mitochondrial electron transport, along with a buildup of oxidative substrates, serves to generate a burst of reactive oxygen species (ROS). In parallel, the accumulated intracellular calcium can induce contracture, and these signal arrays set off a cascade of apoptotic or necrotic events, depending on the severity of the injury (277).

Proteomic approaches have been widely used to investigate the molecular mechanisms that underpin myocardial IRI (278–286), including the extensive extracellular matrix dynamics that occur after MI. The role of the extracellular matrix has been described in the focal injury and border zone with bottom-up shotgun proteomics that revealed discordant regulation of matrix proteins indicative of early and late-stage remodeling (287). Binek et al. (258) characterized ischemic and remote tissues in a pig model at four time points within the first week of ischemia and reperfusion to provide a comprehensive view of remodeling that occurs after reperfusion. Their multiplexed quantitative proteomics approach revealed increased inflammatory processes in the early phases of the ischemic region, which transitioned to alterations that have global implications and involve angiogenesis and cardio-renal signaling processes throughout the postreperfusion period. The remote myocardium was also observed to sustain transient alterations in contractile and mitochondrial proteins (258). This reminds us that the heart is not an organ operating in isolation but rather its modulation can impact the whole body and vice versa.

### 5.5. Myocardial Preconditioning

An interesting corollary to IRI is the discovery of preconditioning as a potential protective strategy. Preconditioning in the heart refers to the phenomenon whereby intermittent periods of sublethal ischemia followed by reperfusion can protect the heart against a subsequent prolonged ischemic episode. This phenomenon was first described in the 1980s and has since been extensively studied (274, 288). Preconditioning can be divided into two types: ischemic preconditioning and pharmacological preconditioning. The latter is further subdivided into development into remote conditioning and postconditioning, where interventions are carried out from nonheart organs and/or immediately after an MI, respectively.

Ischemic preconditioning involves the application of one or more brief episodes of ischemia, followed by reperfusion, before a more prolonged ischemic event that would otherwise induce a greater amount of injury. This can be achieved through transient coronary artery occlusion in animal models. The mechanisms underlying ischemic preconditioning are not fully understood, but they are thought to involve the activation of a variety of signaling pathways and transcription factors, leading to the upregulation and downregulation of protective and proapoptotic genes, respectively. Several studies have used proteomic analyses to investigate the protein expression changes that occur during ischemic preconditioning (167, 289–294). Notably, proteomic approaches have been applied to understand the protective role of secreted multifunctional TRIM family protein (MG53) in IRI. This was one of the initial studies to highlight the role of repairing cell membrane damage that is required for tissue regeneration in the context to the heart. Specifically, this work was able to demonstrate that oxidative stress stimulates a phosphorylation event on protein kinase Cδ that enables the secretion of extracellular MG53 in isolated perfused rodent hearts and in cultured neonatal rat ventricular cardiomyocytes (295, 296). MG53 and its potential role, like other secreted factors, are now recognized to be key aspect of cell-cell communication within the heart and broader and can be a therapeutic target (296). Another study performed a systematic shotgun proteomics analysis on purified bovine inner mitochondrial membrane to define the molecular components of the cardioprotective mitochondrial ATP-sensitive potassium channel and identified KCNJ1 (ROMK) as a key pore-forming unit of this protective complex (294). This channel is a particular focus in the kidneys, because of a known mutation resulting in Bartter’s syndrome, and therapeutics in the form of diuretics are being targeted toward it (297).

Pharmacological preconditioning involves the administration of a protective analyte or drug ahead of an ischemic event. Several agents including opioids, adenosine receptor agonists, and certain anesthetics have been used and may have protective properties. Like ischemic preconditioning, the mechanisms that underlie pharmacological preconditioning are not fully understood but are thought to induce the activation of similar signaling pathways and transcription factors. As an example, proteomics profiling of two pharmacological preconditioning agents, adenosine and diazoxide, in isolated rabbit ventricular myocytes implicates a convergent mechanism of action that involves the altered protein and phosphoprotein expression of mitochondrial energetics (see FIGURE 3). Among these proteins were subunits of tricarboxylic acid cycle enzymes and oxidative phosphorylation complexes (298). Similar approaches were used to identify phosphorylation sites in myosin light chain 1 following adenosine treatment (81), as well as changes in phosphorylation status following diazoxide treatment (299).

### 5.6. IRI-Associated Pathologies

A brief ischemia can cause a transient contractile dysfunction within 24 h after reperfusion defined as a “stunned myocardium.” This pathology leads to alterations of collagen matrix structure; however, the proteome and phosphoproteome changes remain poorly described. A study focused on delineating those regulations in pig models using left anterior descending coronary artery occlusion. Tissue analysis revealed regulation of proteins and phosphorylation sites involved in the downregulation of the contractile function and extracellular matrix remodeling as well as factors involved in programmed cell death (300); however, the key drivers of these processes are not yet fully defined and validated.

Repetitive myocardial ischemia can cause a long-term adaptation named “hibernating myocardium.” LC-MS analysis of a porcine model of hibernating myocardium identified 225 proteins with altered abundance compared with normal healthy myocardium involving many key cardiac pathways such as metabolism, apoptosis, stress response, contraction, cytoskeleton, transcription, and translation (301). Notably, this study highlighted adaptative mechanisms in response to repetitive ischemia through an upregulation of the cardioprotective proteins across many parts of the cell while diminishing the quantity of cardiomyocyte contractile proteins. This suggests that the heart can respond and adapt to the use of the muscle for the preservation of cardiomyocyte viability in the long term. The cardiac proteome of a similar model, analyzed by 2-D DIGE, correlates these previous results, emphasizing the interplay or coordination between the contractile proteins and metabolism potentially initiated by the cellular oxidative stress response (302–304). More specifically, proteomic characterization using a mouse model revealed reduction of the phosphorylation state of myosin light chain 2 and cardiac troponin I, which also correlates with previous observations in porcine models of disease (304).

## 6. SUBPROTEOMES OF THE HEART

Sample preparation techniques hold the key to addressing the broad dynamic range inherent within the cardiac proteome. In its simplest form, dividing lysates into subfractions based on broader biochemical features splits the complexity of a sample and is a particularly useful tool when highly abundant proteins segregate from their low-abundance counterparts. Myofilament proteins are by far the most highly expressed proteins of the cardiomyocytes, which dominate the mass of the heart. This imbalance can lead to an analytical overrepresentation of these proteins and the suppression of coeluting peptide ions derived from other proteins in the cardiomyocytes and other cell types present in heart tissue. Thus, lower-abundance proteins that may be integral to key regulatory and signaling pathways are often analytical victims of the broad myocardial dynamic range. Dividing a sample into relevant fractions with constituent “subproteomes” is a common preparative strategy applied throughout proteomics to address this challenge. There are three broad techniques by which a sample of cells or tissues can be divided into subproteomes: sequential extraction, differential centrifugation, and affinity capture (305). Each of these techniques results in the enrichment of different subcellular components and their constituent proteins and by extension can address specific research questions (95). The separation of a sample into subproteomes, whose ensuing data can subsequently be recombined, has two key benefits: *1*) Subproteomes are individually less complex than lysates and are therefore amenable to deeper and more comprehensive characterization. *2*) The analysis of individual compartments can provide additional context for changes in protein expression or modifications within a given subproteome.

**FIGURE 6.**
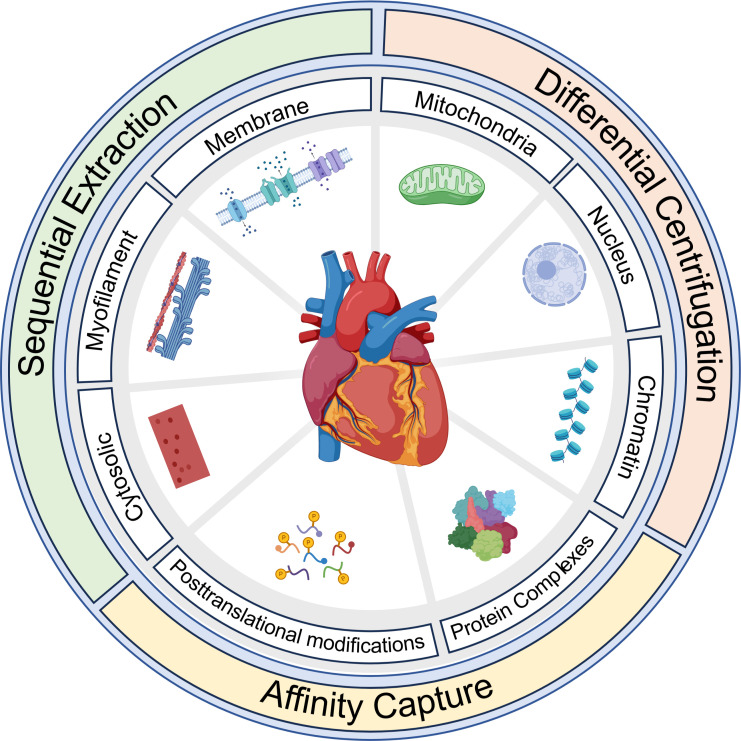
Experimental sample preparation techniques for subproteome fractionation and isolation. Heart tissue/cells can be processed differentially according to the desired subproteome fraction. Sequential extraction may be performed to obtain a cytosolic, myofilament, or membrane fraction. This generally requires a sequential purification of tissue/cell lysates through differential pH levels aided by sample pelleting via centrifugation to obtain the desired fraction. Neutral sample pH will separate cytosolic proteins; acidic pH will purify myofilament-related proteins; and the residual pellet will contain membrane material. Differential centrifugation, often utilizing an ultracentrifuge without altering the sample pH levels, can be used for isolation of mitochondria, nuclei, and chromatin. The desired organelle fractions will separate by mass, allowing for independent removal of desired fractions. Affinity capture and other chemical biology approaches are useful to isolate specific protein complexes and for the enrichment of posttranslational modifications (PTMs). These captures are usually performed in vitro, although several modern labeling techniques [such as azidohomoalanine (AHA) labeling and in vivo crosslinking] may be utilized within living cells. All above methods yield a cellular subproteome, allowing for an increased dynamic range and quality of mass spectrometry (MS) data.

These fractions are typically acquired individually with LC-MS/MS, and the datasets resulting from a sample can subsequently be bioinformatically combined (FIGURE 6). In a simple form, a lysate can be divided into soluble and insoluble fractions. To that end, our group developed an easy method for heart tissue that enriches myofilament proteins while preserving their PTM status (93), whereas previous myofilament enrichment required a comprehensive and laborious myofibril preparation (306, 307), although isolation of just the myofilament/sarcomere has been used for PTM analysis, as discussed in sect. 4. This “IN Sequence” solubilization of cardiac tissue was also amenable to additional sequential extraction of cytoplasmic and membrane proteins (93). The relatively insoluble extracellular matrix (ECM) proteins, along with their extensive cross linking, make their subfractionation especially challenging. Didangelos et al. (308) and de Castro Brás et al. (309) have developed a combined method for the decellularization of the organ/tissue samples and solubilization of the ECM in aorta and heart, respectively. Similarly challenging are membrane-specific proteomes, whose insoluble transmembrane and highly glycosylated extracellular domains require extensive workflows and specific enrichment techniques to maximize subproteome coverage, as reviewed by Lee et al. (310). In a more recent innovative approach to fractionation, Ge and colleagues developed a workflow that utilizes Azo, a photocleavable, MS-compatible surfactant that is specifically geared toward small samples such as cardiac tissue biopsies (311). These innovative chemical methods can similarly be targeted to N-linked glycoproteins, thus enriching the critically important cell surface subproteome ahead of MS analysis (312). Classical cell biology techniques of organellar isolation remain routine tools used to address the dynamic range of the myocardium. These tools, which can target the cytosol, mitochondria, or the nucleus, have been used to separate structures of interest from the bulk of the myofilament proteins (313–315). This enrichment begins by the homogenization of cells or tissues with a buffer solution that disrupts cell membranes. The buffer composition and technique are specifically tailored to release cellular contents while maintaining the structural integrity of subcellular and organellar components, which are subsequently separated by differential centrifugation. This technique can include several rounds of centrifugation with different speeds and durations and can include sucrose gradients to achieve a desired cellular component (316–318). Generally, this approach is used to study mitochondrial, nuclear, and chromatin proteomes.

### 6.1. Mitochondrial Subproteomes

A very common and early approach to subproteomic analysis involved the isolation of cardiac mitochondrial fraction. Given the high energy demand of the heart, mitochondrial dysfunction and cardiac dysfunction are unsurprisingly related in many cardiovascular diseases. Many proteomic studies that characterize cardiac mitochondria tend to do so in the context of whole tissue analysis of specific disease models and phenotypes. However, several groups used early proteomic techniques to specifically target cardiac mitochondria (319–321). Utilizing 2-D gel electrophoresis followed by in-gel digestion and MALDI-TOF analysis, researchers like Kernec et al. (322) identified and monitored changes in 340 cardiac mitochondrial proteins from creatine kinase-deficient mice. Similarly, Taylor et al. (323) used an isolation technique that generated highly purified mitochondria to characterize 615 mitochondrial proteins from normal human heart tissue. Mootha et al. (324) used DDA-MS proteomics to characterize the mitochondrial proteome of the heart in the context of a tissue-specific mitochondrial proteome survey of the mouse. In this study, organelles were isolated by density centrifugation, solubilized, and size-separated by gel filtration chromatography to yield a batch of ∼15–20 fractions that identified 591 mitochondrial and mitochondrion-associated proteins in mouse hearts (324). The subunits of mitochondrial respiratory complexes were identified in heart tissue by researchers who used a combination of one-dimensional SDS-PAGE, two-dimensional isoelectric focusing/SDS-PAGE, and immunoprecipitation in tandem with reverse-phase high-pressure LC (325, 326). This led to the creation of a mitochondria and mitochondrial associated database, MitoCarta, that contains annotated human and mouse proteins with information on mitochondrial localization based on MS data derived from 14 organs, including the heart (327). This type of data is also found in the Human Protein Atlas, based on antibody immunohistochemistry on human hearts, among other data types (328). Mitochondrion-enriched databases are helpful when investigating mitochondrion-associated diseases such as Li et al. (329), who applied iTRAQ to cardiac tissue from a mouse model with a constitutively phosphorylated mutation in microtubule associated protein 4. Importantly, these databases will become even more critical as single-cell RNA and proteomics is carried out in the future, to identify cell and organelle specificity.

Mitochondrial isolates from heart tissues have served as a platform for performing comparative proteomics (330). This approach was exemplified by Chen et al. (331), who provided a rat cohort with or without a high-fat diet for 28 wk (obese) and subsequently analyzed both whole tissue and isolated mitochondria for quantitative lipid content and gene expression, cardiac function, mitochondrial morphology and function, Western blot analysis of specific proteins, and HPLC measurement of adenine content. These techniques revealed that a high-fat diet led to a cardiac hypertrophy phenotype, with dysregulated expression of genes involved in mitochondrial dynamics, decreased mitochondrial DNA, downregulated complex I–III, and impaired citrate synthase and mitochondrial respiration. Most importantly, the mitochondrial proteomic analysis from these hearts provided a molecular basis behind impaired mitochondrial function and dynamics (331). A similar approach involved DDA-based LC-MS analysis of enriched mitochondrial fractions from cultured human induced pluripotent stem cell (iPSC)-cardiomyocytes derived from dermal fibroblasts, cardiac fibroblasts, and adult and neonatal mouse hearts (332). These experiments revealed that developmental increases in oxidative phosphorylation are regulated, at least in part, by the mitochondrial AAA+ protease Lon (LonP1) protease, which could impact the metabolic shift from glycolysis to oxidative phosphorylation and fatty acid oxidation (332). However, LonP1 has multiple functions including proteolysis, chaperone activity, and binding of mtDNA in both the mitochondria and nucleus. It is involved in the response to oxidative stress, heat shock, maintenance of mtDNA, and the regulation of mitophagy (333), suggesting it potentially as one of the linchpins in obesity or potentially in metabolic syndrome (334). In fact, similar effects are observed in skeletal muscle (335).

Mitochondrial functional assessment techniques have been creatively combined with proteomic workflows to link functional responses with molecular-level data. To that end, Shimada et al. (336) utilized a Seahorse bioanalyzer, Langendorff apparatus, and echocardiography to functionally profile hearts and mitochondria in parallel with proteomics from the same sample. These experiments demonstrated that cardiac dysfunction in a mouse model of polymicrobial sepsis is associated with mitochondrial pyruvate dehydrogenase inhibition and impaired pyruvate-fueled oxidative respiration within the heart, which differs from other diseases. This is important, as in septic patients myocardial dysfunction is a primary predictor of poor outcome, which can have mortality rates of 46–70% (337, 338), and the use of cardioprotective clinical approaches is needed.

Native protein interactions can be covalently captured with a small organic cross-linker, whose two reactive functional groups cross-link adjacent amino acid side chains. The resulting adducts can then be mapped with MS-based peptide sequencing. This MS-cross-linking analysis was performed by Liu et al. to map the mitochondrial protein interactome. In sum, this study impressively identified >3,000 protein-protein interactions and covered about half of the mitochondrial proteome (539).

Metabolic labeling is an MS-based technique that was used by Kim et al. (340) to determine the relative turnover rates of mitochondrial proteins. In this study, nine mice were fed with heavy water (D_2_O), and mitochondria were subsequently isolated from cardiac and hepatic tissues at time points spanning from 0 to 90 days. The turnover rates of 458 mitochondrial proteins revealed that median half-life of the cardiac mitochondrial proteome was 17.2 days, whereas that of hepatic tissues was 4.26 days, indicating that organelle protein turnover has a clear tissue specificity.

Proteomics can also be used to study the effects of drugs on mitochondrial protein PTM status. Berthiaume et al. (540) used an antibody against acetylated lysine to enrich for acetylated peptides in hearts of diabetic rats treated with methylene blue. Methylene blue inhibits nitric oxide synthase, reducing nitric oxide (NO), and it is used therapeutically for management of patients with methemoglobinemia such as when they are hypotensive and refractory to vasopressors and fluids after on-pump cardiac surgery. This proteomic analysis shows that methylene blue decreased the aberrant acetylation of mitochondrial proteins in the diabetic heart and improved mitochondrial function by facilitating NADH oxidation (540). Alternatively, Umbrasas et al. (341) used mitochondrial phosphopeptide enrichment to identify changes to the phosphoproteome of the rat heart following treatment with NOC-18, a NO donor. The administration of this nitric oxide precursor is known to be protective against ischemic damage, and its mechanism involves phosphorylation changes to several members of the mitochondrial permeability transition pore, specifically the α-subunit of ATP synthase and adenine nucleotide (ADP/ATP) translocase 1. Thus, phosphorylation is a key PTM involved with the regulation of a key complex involved with both IRI and the maintenance of intracellular redox state (341).

### 6.2. Nuclear Subproteomes

Another cardiac subproteomic option is to isolate the nuclear fraction, as the nucleus is the control center of the cell and contains important regulatory proteins that can impact cardiac function (342). By isolating the nuclear fraction and analyzing its proteome, researchers can identify nuclear proteins that play a role in cardiac function, such as transcription factors that regulate gene expression (94, 343). For example, Franklin et al. (344) used sucrose centrifugation to isolate nuclei from mouse and rat cardiac tissue and used additional fractionation techniques to obtain acid-soluble proteins, chromatin-bound molecules, and nucleoplasmic protein fractions. From these fractions they identified 1,048 nuclear proteins including many histone proteins and transcription factors. This early work has led to the extensive investigation of the cardiac chromatin genome characterizing epigenetic influence in several models of cardiac disease (345–350). Of note, this group identified Smyd1, a histone methyltransferase, to be a key regulator of cardiac energetics by maintaining mitochondrial function though transcriptional control of PGC-1a, which is a master regulator that stimulates mitochondrial biogenesis and regulates many aspects of metabolism (349, 351, 352).

### 6.3. Sequential Extraction

An alternate approach to subproteomic analysis has been the use of sequential extraction protocols to divide the proteome across multiple fractions enriched for proteins of different attributes. One cardiac specific example was the introduction of the IN Sequence cardiac-compatible sequential extraction protocol (247). By homogenizing and centrifugating cardiac tissue in buffer at three different pHs, three distinct cardiac subproteomes can be extracted: a cytoplasmic-enriched extract (neutral pH), a myofilament-enriched extract (acidic pH), and a membrane protein-enriched pellet (93). This method allowed for complete proteomic analysis, divided over three fractions, but sequestered the overrepresented myofilament proteins to their own fraction, providing more in-depth analysis of the other aspects of the myocardium. This method has been used in a variety of cardiac proteomic studies. For example, the IN Sequence protocol was used in a stem cell-treated model of HFpEF, as mentioned above in sect. 5.3 (268). This method was also used to characterize multiple cardiac myofilament protein isoforms in rats (353). The use of high-field asymmetric waveform ion mobility mass spectrometry (FAIMS) has been found to provide similar proteome-resolving power but requires less sample processing and instrument time (178).

Other groups have pursed differing approaches to sequential extraction to obtain enriched fractions. Yin et al. (215) examined the myofilament subproteome in skinned rodent cardiomyocytes treated with endothelin-1 and isoproterenol to induce phosphorylation. In this case, detergents were used to disrupt the cell membrane and remove the cellular contents, leaving the myofilament apparatus intact. They reported characterization of >600 proteins in the myofilament fraction and observed dynamic subcellular localization behavior by a regulatory subunit of protein phosphatase 2A with isoproterenol treatment (215). In a recent publication, a rapid MS-based method for single fibers in skeletal muscle has been published, opening new in-depth ways to map the protein stoichiometry and PTM status of this important subproteome on a large scale (354, 355).

### 6.4. Protein Complex Purification

The interactions that proteins form, both stable and dynamic, are a key determinate for their function. For example, proteins can be arranged in coordinated signaling complexes to facilitate substrate availability or provide inhibition to other members of a complex. The sum of these interactions is termed the “interactome” and represents an additional level of regulation within a cell (356). The cardiac interactome is an essential component for regulating cardiac function. Analysis of the cardiac interactome relies on a variety of techniques including affinity purification, coimmunoprecipitation, and yeast two-hybrid screening. Each of these can provide unique windows through which to understand the molecular mechanisms that underlie cardiac physiology and disease (357–359). Gonzalez-Teran et al. (360) defined the interactome of two transcription factors known to be implicated in congenital heart disease at birth. They hypothesized that mutations in proteins interacting with GATA4 and TBX5 transcriptional complexes could change their associations and alter their regulatory roles. The authors used immunoaffinity capture to target GATA4 or TBX5 and identify their interactome in cardiac progenitor cells. Integrated analysis of the binding partners within known congenital heart disease variants revealed that perturbations of the GATA4-GLYR1 interaction by missense mutation was an important initiating factor of the disease (360).

Beyond genetic disorders of heart disease, interactome dysregulation is also a hallmark of HF. To that end, Chiang et al. (361) investigated the protein associations of Protein phosphatase 1 during the development of HF. They targeted protein phosphatase 1 (PP1) by immunoprecipitation in transverse aortic constriction (TAC)- or sham-treated mice and identified its associated binding partners. They observed >70 binding partners, 9 of which had altered interactions during the progression of HF, 1 of these being Ppp1r7, which binds directly to the catalytic domain of PP1 (361, 362). A similar investigation was done for O-GlcNAc transferase, which is a key signaling enzyme that posttranslationally modifies proteins by catalyzing O-linked *N*-acetyl-β-d-glucosamine (O-GlcNAc) moieties to serine and threonine residues of cytoplasmic, nuclear, and mitochondrial proteins. The authors found >130 proteins in the O-GlcNAc transferase interactome, including host cell factor 1, which demonstrated a time-dependent dissociation after oxidative stress (363). O-GlcNAc is an intercellular glycosylation and major sensor of metabolism by modulating protein function (see for review Refs. 364–366). As tools have been developed, primarily by MS, the number of identified sites has increased exponentially, leading to a broader understanding of the breath of this PTM’s impact (see, e.g., Refs. 367–370).

The interactome of ryanodine receptor type 2 (RyR2) was similarly studied and found to be altered by its phosphorylation status. RyR2 is phosphorylated at serine 2814 in both HF and atrial fibrillation. An affinity approach revealed differential interactions between wild-type, unphosphorylated, and constitutively phosphorylated mutants of RyR2, including the mitochondrial isoforms of Isocitrate dehydrogenase β and Apoptosis-inducing factor 1 (371). Another study evaluated the structural scaffold 14-3-3 protein, which consists of several isoforms with potential different functions, and revealed 52 interacting proteins in the heart, localizing it to the mitochondria (372). As the authors point out, there are a number of 14-3-3 inhibitors and stabilizers that are being tested as therapeutic in systems other than the heart (373).

### 6.5. Affinity Capture and Protein Proximity Labeling

Subproteomes can be alternatively analyzed by targeting of a group or class of proteins that have common associations or characteristic. In practice, this is most applicable to the analysis of protein complexes or posttranslationally modified proteoforms. A variety of affinity capture approaches are available to isolate and enrich these subproteomes. For proteins that interact with one another, capture techniques include the widely used coimmunoprecipitation, in which a capture immunoglobulin binds a specific “bait” protein with a view toward the secondary capture of its binding partners. In the case of PTMs, subproteomes can be enriched by physicochemical affinity chromatography or by affinity tags with specific chemistries that adhere or capture common chemical moieties. Each of these strategies is applied to study of cardiac biology and disease.

In addition to characterization of the interactome with immunoaffinity techniques, interactions can also be studied with proximity labeling strategies (374, 375). In this strategy a target protein is recombinantly fused with a promiscuous biotin labeling protein. Upon initiation, proteins near the target protein are selectively biotinylated within a living cell. There are a few versions of the basic strategy, each using different mechanisms of labeling, APEX, BioID, BioID2, TurboID, or split TurboID (376–378). For example, BioID utilizes a mutant form of the *Escherichia coli* biotin protein ligase BirA that is engineered to indiscriminately biotinylate any protein within a small radius, whereas APEX uses an engineered ascorbate peroxidase to generate short-lived biotin-phenoxyl radicals in the presence of H_2_O_2_ to label proteins close by. These biotinylated proteins can then selectively be captured with streptavidin and identified by using MS to characterize interacting protein complexes (75, 379). The APEX proximity labeling approach was combined with stable isotope labeling to reveal the interactomes of caveolin 1 and 3 in rat neonatal cardiomyocytes. Caveolin 3, which is associated with arrhythmogenic cardiomyopathy, was found to specifically interact with the monocarboxylate transporter McT1. Further analysis showed distinct distributions of the caveolin complexes and a stabilizing role for McT1 in preventing arrhythmogenic late sodium currents (75, 357, 380).

Li et al. (381) used BioID2 proximity labeling to define the architecture of the adherens junctions that connect cardiomyocytes and transmit the mechanical forces of contraction. By characterization of the interactome of N-cadherin in mouse neonatal cardiomyocytes, >350 interacting proteins in these junctions were identified (381). The interactome of the inward rectifier potassium channel (K_ir_2.1) was similarly defined. This channel complex, a key regulator of the cardiac action potential, was subjected to proximity labeling to identify 218 interacting proteins. Among these interacting proteins, PKP4 was found to modulate K_ir_2.1 currents (382). Proximity labeling continues to be a powerful technique for identifying and characterizing the myocardial interactome and for elucidating those signaling pathways and molecular mechanisms that underlie cardiac development, function, and disease. This strategy has also been employed in zebrafish hearts. BioID2 was used to track dynamic interactions of ErbB2 in a model of heart regeneration (383). The authors observed RhoA to be an interactor of ErbB2, and blockade of their interaction inhibited muscle regrowth. This study provides excellent support for in vivo characterization of dynamic interactions.

## 7. POSTTRANSLATIONAL MODIFICATIONS AND CHEMICAL DIVERSITY OF PROTEINS

**FIGURE 7.**
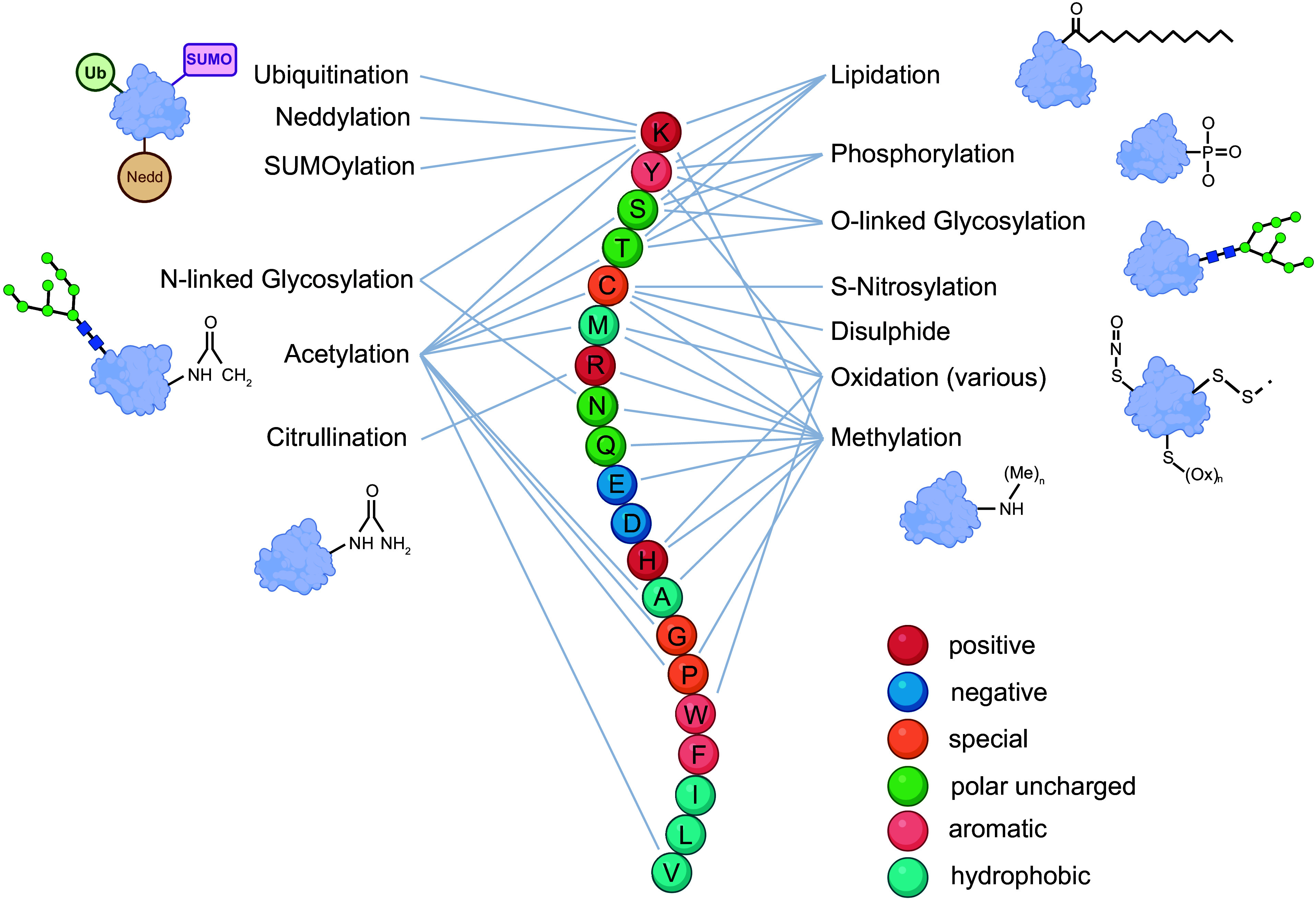
Diagram of possible posttranslational modifications (PTMs) and their sites of incorporation. Commonly represented PTMs are portrayed here with lines linking them to their cognate residues of installation, denoted by the 1-letter amino acid code. Color scheme of residues refers to the different residue types. Representation of several PTMs in diagrams can additionally be found, highlighting the important structural differences between polypeptide-based PTMs (ubiquitination, neddylation, etc.); oligosaccharide-based PTMs (N-linked glycosylation, etc.); and chemical modifiers as PTMs (methylation, oxidation, etc.). SUMO, small ubiquitin-related modifier.

In addition to transcription and translation, protein PTMs represent a vast, diverse, and dynamic mechanism through which protein activity is regulated and fine-tuned. PTMs are broadly defined as the addition to or removal of chemical groups from a protein, yielding an altered proteoform that may or may not have a functional consequence for the modified protein (384). PTMs often fractionally affect a population of a given protein or protein family in response to a stimulus and can serve to functionally enhance or suppress signal transduction or protein function (385). Phosphorylation is the best-known and best-understood PTM because its PO_4_^−2^ moiety induces a charge difference that was more easily detected by less sophisticated analytical techniques such as 2-D gel electrophoresis. Although important, phosphorylation is far from the only PTM, and this list includes modifications with an array of chemical characteristics with specificity toward a large number of amino acid residues (FIGURE 7). This diversity, along with the context dependence of PTMs, represents a significant challenge. Furthermore, most polypeptides contain multiple PTM acceptance sites, and many can simultaneously accept multiple types of PTMs. Phosphorylation-dependent intracellular signaling is highly dynamic, in constant flux, and responsive to cellular changes. Phosphorylation also works in concert, in tandem, or in competition with other PTMs such as O-GlcNAcylation, which similarly affects serine and threonine residues (386). Thus, the complexity of data analysis ramps up exponentially when proteomics experiments are designed to consider PTMs. Unsurprisingly, there is a constant appetite among researchers for novel bioinformatical tools that can accommodate, process, and visually represent the datasets derived not only from proteoforms of single proteins but also from proteoforms vertically integrated within global networks of context-dependent cell regulation. It also has to be recognized that there are many PTMs with limited tools for investigation and hence their role in the heart is not known. Over the next decade, as additional tools are developed or existing ones improved, we will be able to bring to light these underappreciated aspects of the proteome.

### 7.1. Phosphorylation

**FIGURE 8.**
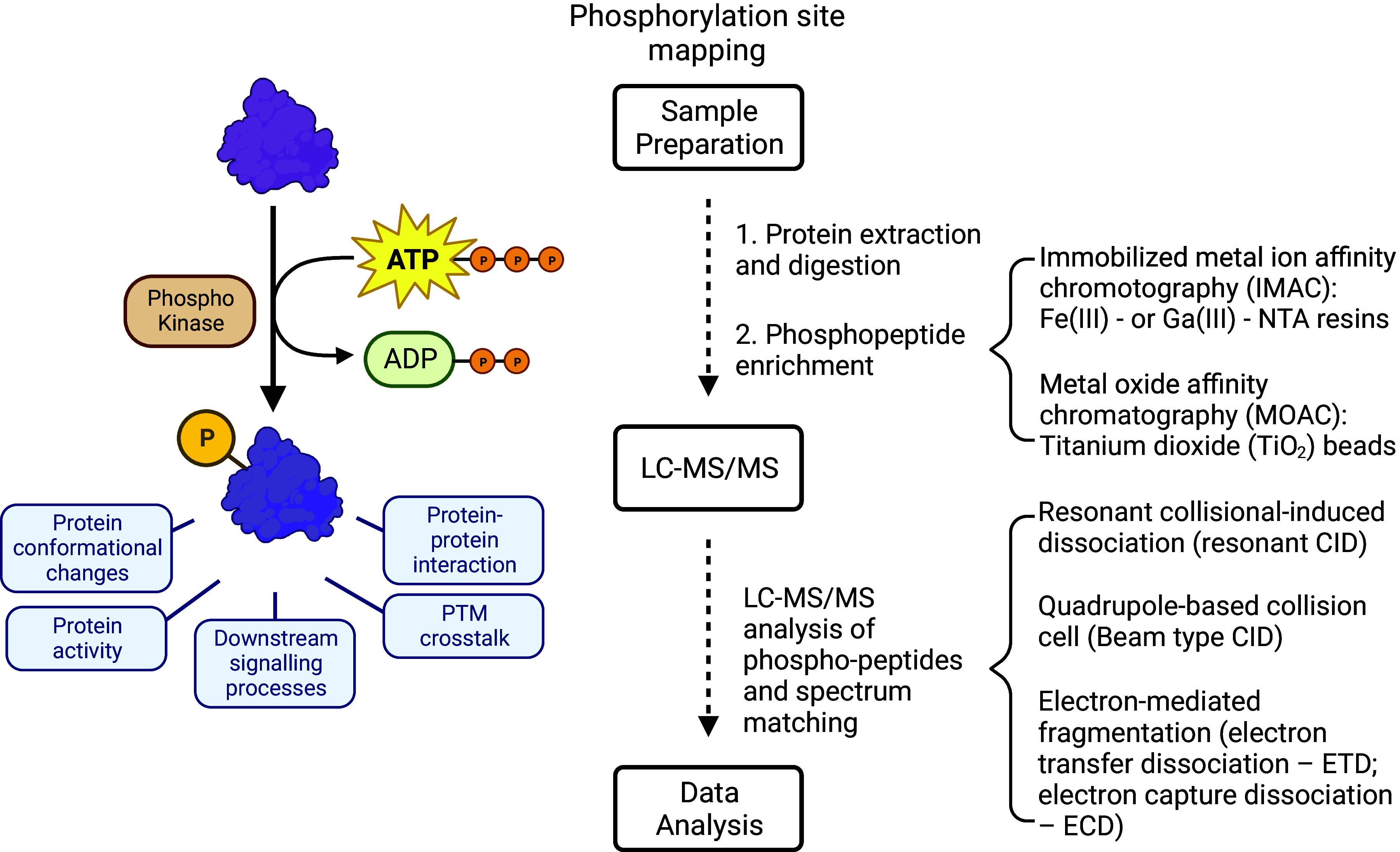
Common phosphopeptide enrichment and fragmentation strategies for mass spectrometry (MS)-based analysis of the phosphoproteome. A typical ATP-dependent protein phosphorylation schematic can be viewed on *left* with associated downstream effects. Within sample processing, a common step for phosphopeptide enrichment is immediately following tryptic digestion steps. These typically involve affinity metal capture techniques. A range of MS-based dissociation and peptide enrichment strategies can be additionally carried out in subsequent steps to increase phosphopeptide purity. LC-MS/MS, liquid chromatography-tandem MS; NTA, nitrilotriacetic acid; PTM, posttranslational modification.

Kinase-based phosphorylation is a well-characterized mechanism of intracellular signal transduction. The addition of the negatively charged phosphate to the side chain hydroxyl group of a serine, threonine, or tyrosine can alter the structure and therefore function of a target protein in a variety of ways including activity, interactions, and subcellular localization (FIGURE 8) (387–390). When considering phosphorylation changes of a single protein between sample groups, it is often useful to consider direct and indirect upstream effectors and regulators of signal transduction in the form of kinases and phosphatases. Often, these pathways converge on major protein regulators of signal transduction (JNK, ERK1/2, p38, p53, JAK/STAT, etc.), many of which have a broad array of substrate proteins and act across multiple intracellular signaling pathways (391). Despite their numerous and sometimes divergent substrate and partner proteins, these major protein regulators operate in defined phenotypically relevant signal transduction pathways that are broadly described by their end result (proinflammatory, proapoptotic, cell growth proliferative or inhibitory, cell differentiation, etc.), and these can be critical to understanding the significance of differential protein phosphorylation.

Most investigations to characterize a proteome’s phosphorylation status have included a phosphopeptide enrichment protocol (392–397). Enrichment was traditionally primarily performed using immunoaffinity, where antibodies are raised against the phosphorylated Ser/Thr or Tyr epitope and used to enrich phosphor peptides from digested lysates (398, 399). Although this approach can be effective, it has largely been supplanted by immobilized metal or metal oxide affinity chromatography (iMAC or MOAC) (400, 401) (FIGURE 8). In these approaches a digested lysate is applied to a column containing a positive transition metal ion, usually Fe(III), Ga(III), or TiO_2_ (402, 403). The negatively charged phosphate groups are attracted to and retained on the column by the positive anions. After peptides are enriched, the labile nature of the phospho group can make identifying the phosphorylated residues difficult. Early attempts to fragment phosphorylated peptides often resulted in a neutral loss of the phospho group, leaving the remaining peptide intact. This has been addressed by using alternate forms of “softer” fragmentation to target the peptide backbone. Phosphopeptides have been fragmented by resonant collision-induced dissociation (CID) or electron-mediated fragmentation using electron transfer or capture dissociation (ETD or ECD) (404, 405).

Phosphoproteomic approaches have been used across the spectrum of heart diseases to provide molecular-level insights that underscore physiological changes, and MS workflows and techniques are increasingly available that are specifically designed to quantify cardiac phosphoproteins (185). Phosphoproteomics have identified key differences between ischemic and nonischemic HF, finding differentially phosphorylated substrates of the serine/threonine protein Casein kinase (Ck2) in ischemic HF that were reciprocally dephosphorylated in nonischemic HF (406). Ck2 was also found to play a key role in phosphoprotein differences involved in cardiac dyssynchrony (407). Hypertension is often seen as a major comorbidity of HFpEF (228), and Valero-Muñoz et al. (408) used a mouse model of hypertension to study the proteomic and PTM signatures associated with HFpEF. Their study revealed proteins associated with mitochondrial function and oxidative metabolism to be both differentially expressed and distinctly phosphorylated and acetylated in HFpEF. The authors further observed that Titin, specifically within its N2B isoform, was differentially phosphorylated in fractions of the diseased left ventricle (LV). This finding implicated Titin hyperphosphorylation at the Z-disk binding region with muscular stiffness (408).

The myofilament protein Titin is the largest protein in the human body, and although its peptides are often overrepresented in myocardial bottom-up MS experiments, its proteoforms have important physiological functions. Titin hyperphosphorylation, mediated by proline-directed kinases such as ERK1/2 and Cdc2, is a proteomic hallmark in HF (409). Reciprocally, Troponin I (TnI) and myosin binding protein-C (MyBP-C) phosphorylation were significantly decreased in cells harvested from patients experiencing dilated cardiomyopathy in association with truncated titin mutant variants (410). Other signal transduction pathways within cardiomyocytes that have been highlighted via PTM-based studies include ERK1/2, protein kinase A (PKA), and, most recently, the Rho kinase signaling pathway (411). It is a common theme with phosphoproteomics to uncover extensive phosphorylation, including new sites that were additionally reported in the DSS HFpEF model (268) and human heart tissues (183), revealing several previously published phosphosites being quantitively minor compared with the newly uncovered ones.

Mouse models with an R9C phospholamban mutation have served as a useful platform of dilated cardiomyopathy, which is a key contributing factor to HF. One study used this murine model of dilated cardiomyopathy to map 7,589 phosphopeptides within 1,848 cardiac proteins, finding altered phosphorylation of ubiquitin-related complexes involved with the Notch-1 transmembrane receptor processing (412, 413). The observed downregulation of this receptor, which is involved in cell fate decisions, was contributing to cardiac cell dysfunction, and was supported by later experiments utilizing Notch-1-specific molecule inhibitors (412). Xu et al. (414) focused on phosphotyrosine modifications in their characterization of mouse models of cardiac hypertrophy. Among several observations, the authors observed reduced pTyr in the myofilament proteins and activation of the epidermal growth factor receptor pathway in hypertrophy (414). Phosphopeptides can additionally contribute to the characterization of proteins, lending the ability to perform subsequent mutagenesis experiments that alter a polypeptide’s structure and provide context to understanding the function of a particular phospho-accepting site (415).

Phosphorylation plays a key role in regulating mitochondrial protein turnover. HF induces a global, general dephosphorylation of mitochondrial proteins, which impacts intracellular transport and metabolism-related ontologies (416). The dynamic networks involved with mitochondrial phosphoproteomic reprogramming play a role not only in models of cardiac disease but also in exercise experiments within extension of life span studies (341, 417). Phosphorylation-based signaling is suppressed or changed by specific phosphatases. Although these enzymes are less abundant and less specific to phosphoprotein substrates compared with kinases, they are more challenging to study with established biochemical methods (362). The combination of broader proteomic techniques with PTM analysis offers a robust platform for investigations into the role of phosphatases and has yielded lists of substrates and interactors of major cardiac phosphatases (83).

Changes to different PTMs can occur in tandem with one another, but understanding this interplay can prove to be experimentally challenging because their analysis often involves tailored isolation and preparative workflows. Nevertheless, Koser et al. (418) used a general phosphoproteome and acetylome approach to characterize differences between Zucker diabetic and spontaneous hypertensive heart failure (SHHF) rat models of HFpEF. They observed proteoform signatures consistent with systemic inflammation and endothelial remodeling, with significant changes to the acetylome as it pertained to metabolism, energy production, and contractility (418). Similar results were found in earlier phosphoproteomic studies of SHHF rats (419, 420). We also expect that as more PTM-based tools become available it will be easier to characterize the interplay/cross talk between PTMs and determine the functional consequences of different PTMs.

### 7.2. Acetylation

Protein acetylation is an expanding field owing to improvements in sample purification and dedicated MS acquisition techniques. Generally, acetylation falls under the larger, well-recognized umbrella of dynamic modifications known as acylation. Examples of acylation events include acetylation, formylation, butyrylation, crotonylation, myristoylation, palmitoylation, and a host of other fatty acid modifications. Acylation events can modify methionine, serine, glycine, alanine, threonine, cysteine, valine, and lysine residues and occur in an estimated 80% of all eukaryotic proteins (421).

Acetylation has historically been studied as modifications to histone structural complexes to induce reorganization ahead of transcriptomic and epigenetic events. The dynamics of histone acetylation by histone acetyltransferases (HATs) and deacetylases (HDACs) has a long history in the cardiac field. Literature accounts suggest that HDAC inhibitors in cardiac tissue can decrease cardiac fibrosis and reverse atrial arrhythmia inducibility, cardiac hypertrophy, and adverse hypertrophy linked to cardiac pressure overload (422–424). Thus, HDAC inhibition may confer a cardioprotective effect within cellular models of heart disease (425–428). Within cardiac studies, lysine acetylation has been implicated in a range of intracellular processes as the underlying mechanism behind cardiac tissue remodeling events (429). In one of the earliest in-depth acetylation proteomics studies, Foster et al. (430) used a guinea pig model of HF and identified nearly all cardiac protein lysine acetylation events related to fatty acid metabolism and oxidative phosphorylation, with mitochondrial proteins representing 59% of identified proteins and 64% of identified sites. The nuclear transcriptomic regulators E1A (p300) and CREB binding proteins, as well as a number of myofilament-related proteins and the intracellular Ca^2+^-regulatory proteins RyR2 and SERCA2, were additionally identified as substrates for acetylation. These findings suggest that acetylation of cardiac proteins predominantly affects proteins involved in energy metabolism and transcriptomic global events (431), potentially working on different timescales of cell regulation.

It is now clear that acetylation of nonhistone proteins is an important regulatory event of cell function; beyond transcriptional regulation, it also alters enzymatic catalysis and protein signal transduction and is crucial for homeostatic cellular activity. One of the first studies to highlight global protein lysine acetylation modifications showcased 3,600 lysine acetylation signatures across 1,750 proteins driving change in a broad number of cellular processes (432). Similarly, Horton et al. (433) applied tandem mass tag (TMT) labeling to study the acetylome of both mouse and human failing hearts. This approach revealed that lysine hyperacetylation of the mitochondrial protein succinate dehydrogenase A (SDHA) decreased its catalytic function and impaired complex II-driven respiration. This protein is a component of the tricarboxylic acid (TCA) cycle, and its dysregulation converges on a HF phenotype (433). The impact on acetylation and mitochondria has a long history, including a study published in 2005 that demonstrated the ability of time-of-flight MS to yield a list of acetylated proteins from cardiac tissue and identified a number of potential biomarkers and serum-based polypeptides (106). These acetylated proteins were predominantly mitochondrial and additionally included ATP synthase, long-chain acyl-CoA dehydrogenase, creatine kinase, malate dehydrogenase, and pyruvate dehydrogenase, although the exact effect of acetylation on protein function or the downstream phenotypic outcomes remains largely elusive (434).

Mitochondrion-based acetylation within cardiac tissues unsurprisingly involves proteins related to cellular bioenergetics and fatty acid metabolism. Accordingly, cyclophilin D (CypD) is an isomerase that is central to mitochondrial metabolism and permeability transition. Its deletion led to widespread mitochondrial protein acetylation and created unfavorable Ca^2+^ and NAD^+^/NADH levels within the organelle. These changes are thought to induce hyperacetylation as a compensatory mechanism to combat apoptotic processes (435). The idea of compensatory mitochondrial hyperacetylation is supported by Carnitine acetyltransferase (CrAT) and Sirtuin 3 (Sirt3) knockout studies, which are also characterized by intramitochondrial hyperphosphorylation in mouse models of cardiac pressure overload and HF (431). Sirt3 plays a key role in mitochondrial deacetylation, but aside from extensive hyperphosphorylation one study did not find any noteworthy metabolically relevant phenotypes in Sirt3^−/−^ knockout mice (436). In contrast, other proteomic investigations indicated that the increased mitochondrial acetylation derived from Sirt3 deficiency in cardiac tissues impaired contractile function and increased myocardial fatty acid oxidation (437, 438).

Furthermore, acetylation being a regulator for mitochondrial protein activity was additionally observed in rat livers and hearts. Here, acetylation increased the activity of isocitrate dehydrogenase type 2 (IDH-2) but decreased the activity of Glutathione peroxidase 1 (GPx), with overall improved mitochondrial ROS profiles and improving cellular viability (439). Furthermore, GPx deacetylation in cancer cells decreased NADPH-to-NADP^+^ and GSH-to-GSSG ratios [which can induce oxidative PTMs (OxPTMs)], suggesting a potentially viable deacetylation-based therapeutic intervention for targeted cell death (439)

### 7.3. Oxidative Posttranslational Modifications

**FIGURE 9.**
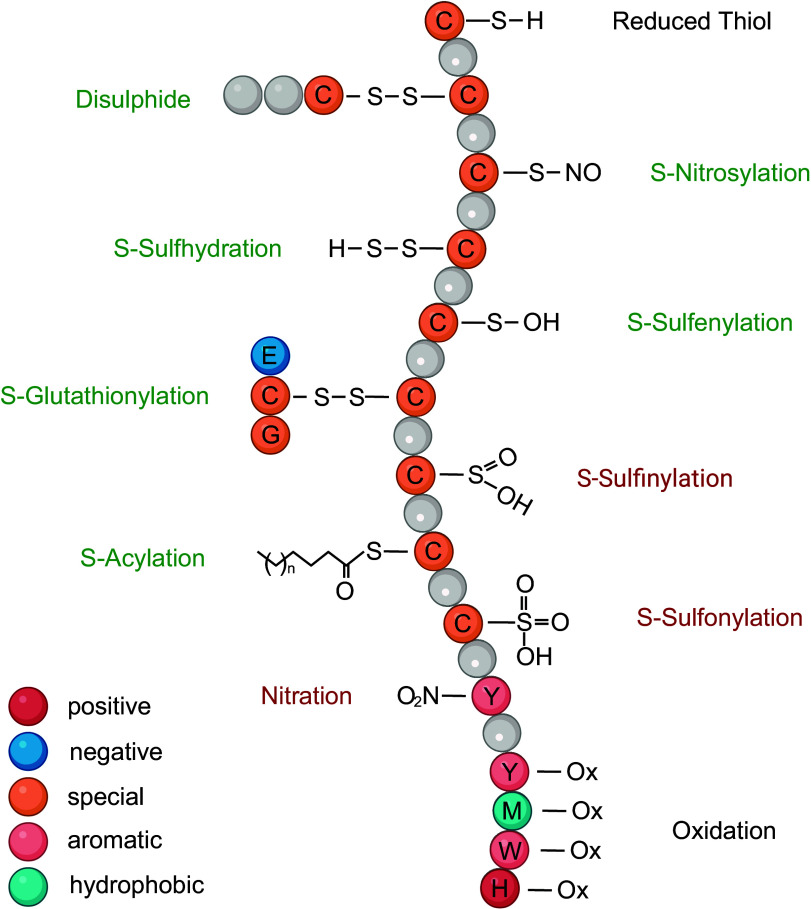
Survey of common oxidative posttranslational modifications (OxPTMs) and the amino acid residues they modify. The chemistries and names of many possible OxPTMs are portrayed here. Names of reversible modifications are listed in green; irreversible modifications are listed in red. Color coding of amino acid residues is denoted in the key and is the same as in FIGURE 7.

Beyond phosphorylation and acetylation there are a host of other PTMs that have been found to have regulatory implications for the heart in health and disease. One class of PTMs that has gained appreciation over the last 25 years is oxidative modifications (OxPTMs) in response to a shifting redox balance in the cell (FIGURE 9) (440). Reactive oxygen and nitrogen species (ROS/RNS) can act as both physiological second messengers and agents of pathological oxidative damage depending on the cellular context. A prime example of the dual nature of ROS/RNS can be found in the modifications of cysteine thiol groups (441). Physiological fluctuations of ROS/RNS can reversibly modify selective reactive cysteines, known as redox switches, to trigger signaling events, whereas excess ROS/RNS can accumulate irreversible modifications with negative effects on function (442, 443).

Proteomic characterization of some of the reversible modifications has been difficult because of their labile nature and challenging chemistry, making traditional MS approaches ineffective at identifying these subproteomes. An early solution to identifying labile modifications like S-nitrosylation (SNO) or S-sulfenylation (SOH) was the biotin switch protocol introduced by Jaffrey et al., where target modifications are selectively replaced by a stable biotin group that allows for enrichment (444, 445). Since then, there have been several iterations of this technique to include other modifications, improve specificity, and add quantitative features to this MS assay (446–452). In an interesting technical comparison, Chung et al. (448) used different Cys-reactive quantitative TMT tags (pyridyldithiol or iodoacetyl based) in a version of the biotin switch assay to reveal a context-dependent difference in thiol reactivity to the reagents improving the SNO proteome coverage. An alternate approach for SNO identification is the use of organomercury reagents that can selectively react with reduced Cys and SNO modifications. Once bound to an organomercury resin, proteins or peptides can be released for MS analysis (453).

The SNO proteome has been investigated in the heart in several contexts. One of the early investigations was to understand mechanisms of cardioprotective preconditioning following brief periods of ischemia and reperfusion. In a series of proteomic studies, SNO sites were characterized in preconditioned myocardium, indicating a broad mechanism for protection against further oxidative damage (292, 452, 454–456). This work has been extended to evaluate endogenous sex-dependent differences in cardioprotection (457–459). Another group also investigated cardioprotection via exogenous treatment with nitrite in conjunction with ischemia. By quantifying the SNO proteome, they found significant increases in SNO-modified proteins in the protected state, particularly among mitochondrial proteins (460).

SNO modifications have also been characterized within subcellular compartments of the cardiomyocyte. Figueiredo-Freitas et al. (461) found that treatment of isolated myofibrils with a SNO donor reduced sensitivity to Ca^2+^ and found actin, myosin, myosin-binding protein C, and troponin C proteins to be SNO modified. A similar approach was applied to isolated cardiac mitochondria from rat (462). The SNO proteome was also investigated in heart tissue of a mouse model of Duchenne muscular dystrophy (DMD) (463). The authors investigated the effect of nitric oxide synthase delocalization in the absence of dystrophin and the role of the Trpc6 channel in regulating nitrosative stress. They found reduced SNO in DMD but in a further knockout of Trpc6, as cellular SNO levels were increased, and cardiac function was improved.

Lau et al. (464) performed a meta-analysis of studies where SNO sites were identified in mouse heart by a variety of different proteomic approaches. Despite the heterogeneity in model system and approach, they found a core group of SNO sites common to each of the studies regulating proteins involved in glycolysis, TCA cycle, oxidative phosphorylation and ATP production, long-chain fatty acid β-oxidation, and ketone and amino acid metabolism (464). This type of post hoc comparison is a helpful barometer for the field given the diversity of approaches available to characterize the SNO proteome.

There has also been significant development in the targeting of S-sulfhydration (SSH), S-sulfenylation, S-sulfinylation, and S-sulfonylation through advanced chemoproteomic probes to target thiol-based redox biology and oxidative stress (465). Of note, selective labeling of SSH presents an exquisite challenge to distinguish these modifications from more standard disulfide bonds. Zivanovic et al. (466) used a dimedone switch method that leverages unique activation of sulfhydration with a blocking reagent so that the inner sulfur atom can be preferentially targeted by a nucleophile, in this case, DCP-biotin. Once labeled with a stable group, proteins can be digested and modified peptides captured for MS analysis. A study combining proteomic characterization of SSH and SNO found an additive protective effect when ischemic tissue was treated with H_2_S and SNO donors. They found the increases in SSH- and SNO-modified proteins with H_2_S but a further increase in SNO proteins when the SNO donor was added, suggesting role for each modification with SNO driving the magnitude of the response (467).

There have also been several studies characterizing the interplay between reversible and irreversible oxidative modifications S-sulfinylation and S-sulfonylation (468–470). In a recent study, Rookyard et al. (471) performed a comprehensive proteomic characterization of the reversible cysteine OxPTMs as well as identification of irreversibly oxidized cysteine peptides (Cys-SO_2_H/SO_3_H) in a rat model of ischemia-reperfusion. They identified over 4,000 reversible and 200 irreversible sites and by treating with an aminothiol antioxidant assembled a list of cysteine residues that are susceptible to ROS damage during ischemic injury (471). A similar study was performed during the progression of cardiac hypertrophy. The authors of this particular study were able to characterize the spectrum of oxidative modifications in an isoproterenol-induced mouse model of hypertrophy. They found four pathways to be significantly impacted by redox-modifications, with three in the early phase of hypertrophy whereas glucose metabolism enzymes were impacted in the later stages (472). Their analysis provided a new perspective on the temporal role of oxidative modifications in the progress of hypertrophy.

Amino acid residues other than cysteine can additionally receive oxidative modifications. The reaction of peroxynitrite, a reactive nitrogen species, can produce nitration of tyrosine residues. This physiologically irreversible modification has been found to regulate the function of several target proteins (473–478). Proteomic analysis of nitrotyrosine proteins has been accomplished mainly through immunodetection coupled with MS, as well as through chemical derivatization approaches (479–481). In the heart, tyrosine nitration has been investigated most within the mitochondria. Tatarkova et al. (482) used a Langendorff protocol to induce ischemia-reperfusion injury in rats. Proteomic analysis of the nitrated proteome revealed modification to proteins involved in electron transport, ATP synthesis, fatty acid oxidation, and the tricarboxylic acid cycle. Similar results were obtained from mitochondria isolated from beef hearts treated with peroxynitrite (483). Wang et al. (484) also used a proteomic screen to identify tyrosine nitration sites in mitochondria of db/db mice. They observed two sites of nitration in succinyl-CoA:3-oxoacid CoA-transferase, an enzyme involved in ketolysis. Further functional analysis found that modification of Tyr 4 and 76 significantly inhibited succinyl-CoA:3-oxoacid CoA-transferase activity (484). Although the oxidative PTM is sometimes challenging to isolate and analyze, the evaluated literature portrays that complex oxidative PTMs confer several critically important roles within cardiac cell function in health and disease.

### 7.4. Ubiquitin and Ubiquitin-like Modifications

Ubiquitin (Ub) and the homologous Ubiquitin-like PTMs have an important role in regulating protein function. Ub is the best-known modifier of this group, but since its initial identification several other small-protein PTMs have been identified, including small ubiquitin-related modifier (SUMO) family members, neuronal precursor cell-expressed developmentally downregulated protein 8 (NEDD8), and interferon-stimulated gene 15 (ISG15) to name a few (485, 486). Although these modifying proteins are distinct, they share some structural and mechanistic characteristics. Ub modifies target proteins though a covalent bond to a ε-amino group of a lysine reside. Modification occurs via a multienzyme cascade where Ub is passed from activating (E1) to conjugating (E2) and then to ligating (E3) enzymes before reaching its final target substrate. Ub modification can include Ub itself, to create linear or branched poly-Ub chains linked to a substrate protein (487). Ubiquitinylation is usually associated with marking proteins for degradation via the proteosome, but Ub-like PTMs can have a variety of effects to modulate a protein’s structural and functional properties. This class of PTM has been implicated in regulating cellular processes like proliferation and differentiation, autophagy and mitophagy, transcription, signal transduction, proteolysis, and protein synthesis (488).

A variety of approaches have been utilized to characterize the Ubiquitin-ome, mostly relying on antibody/capture and tagging methods coupled with MS to enrich and identify Ub-modified proteins (489). This has included adding affinity tags to the sequence of Ub or using an antibody to capture Ub and any proteins that are covalently bound to it. Other groups have leveraged Ub binding domains to enrich Ub-modified proteins (E3 Ub ligases, deubiquitinating enzymes, and Ub receptors) (490, 491). By extension, the tandem-repeated Ub-binding entities (TUBEs) approach increased affinity by doubling the binding domains for capturing Ub-proteins (492–494). The tandem approach preferentially enriches polyubiquitylated proteins but has reduced affinity for monoubiquitylated proteins, which can represent up to 50% of the Ub-modified population (495).

**FIGURE 10.**
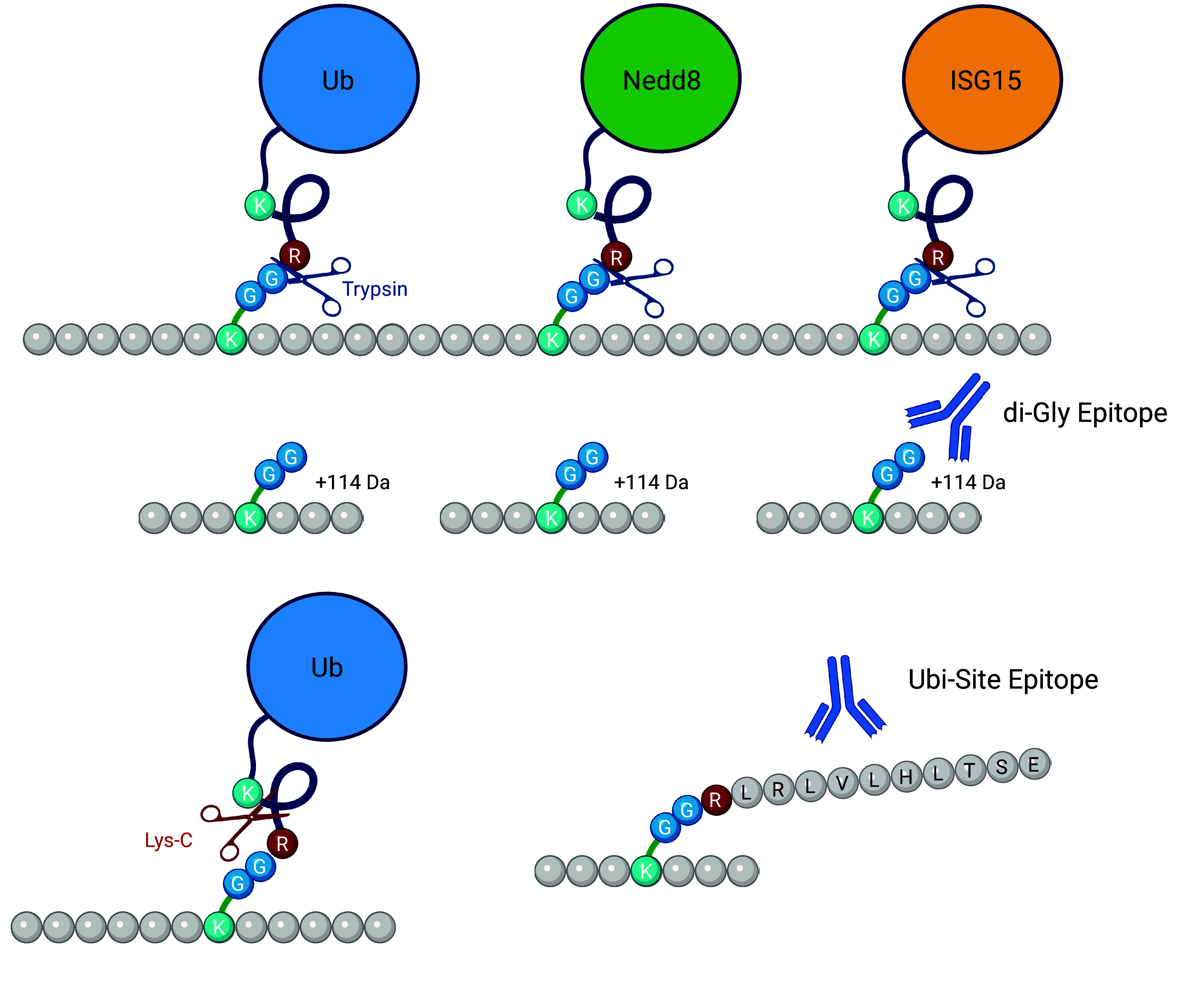
Various approaches for the identification of the Ubiquitin (Ub) and Ub-like associated proteins. Ub and Ubi-like modifiers such as NEDD8 and ISG15 are posttranslational modifications (PTMs) that confer different biological outcomes for their associated proteins upon binding, as showcased at *top*. However, the use of a trypsin-based digest during mass spectrometry (MS) sample preparation leaves an identical diGly motif from all 3 of the aforementioned PTMs. These diGly motifs can be purified with a specific antibody; however, discerning between the 3 PTMs becomes impossible. The use of Lys-C as a protease creates a longer Ub peptide and lends the ability to subsequently use a purifying antibody against the extended Ub peptide, separating out proteins with their specific PTMs.

There are also techniques that target the Ub modifications at the peptide level. During trypsin digestion, Ub substrate proteins and Ub are cut after lysine and arginine within their polypeptide sequence. This leaves a Gly-Gly motif (diGly) on each Ub-ligated lysine residue that can be identified by MS as a site of modification. The currently most popular of these techniques utilizes an anti-K-ε-GG antibody-based immunoenrichment of peptides with the diGly motif (496, 497). However, an interesting challenge in this approach is that NEDD8 and ISG15 modifications have the same diGly motif remaining after tryptic digestion of proteins, making these three modifications indistinguishable during MS analysis because of mass similarities (FIGURE 10). A further refinement of the peptide capture has been the Ub-site antibody enrichment. In this case Lys-C digestion is used to cut only after lysine residues, leaving a 13-amino acid section of the Ub COOH terminus on the substrate peptide (498). An antibody recognizing the longer remnant sequence is used to then specifically enrich the modified peptides, although additional digestion is recommended before MS analysis (492).

In the heart, some of these approaches have been used to characterize the Ub and Ub-like subproteomes. In 2007, Jeon et al. (499) used a transgenic mouse expressing an affinity-tagged Ub to identify 121 modified proteins in the heart including many mitochondrial proteins. In 2023, Wu et al. (541) characterized the Ub-ome in patients with atrial fibrillation. Using a Ub-peptide enrichment technique, they quantified >4,500 sites of modifications in patients with atrial fibrillation or normal sinus rhythm, thereby characterizing the dynamic Ub-ome. Beyond Ub, Hotz et al. (284) investigated changes of the Ub-like PTM SUMO after ischemia-reperfusion (I/R) injury in mice. Using a monoclonal antibody enrichment approach targeting SUMO1 and SUMO2 modifications, the authors identified 100 SUMO1, 50 SUMO2, and 35 shared modified proteins. The study observed dynamic changes in SUMO modification to several components of the ubiquitin proteasome system during cardiac I/R, suggesting modulation of proteostasis during hypoxic stress (284).

### 7.5. Citrullination

Citrullination is a more recently recognized PTM that involves the irreversible peptidylarginine deiminase (PAD) enzyme-mediated conversion of an arginine residue within a folded protein to a citrulline by an imine group removal. Citrullination has been implicated in a number of human diseases including neurodegenerative disorders, cancer, and cardiovascular diseases (500–504). Within the cardiac context, elevated PAD enzyme expression and increased citrullination events were observed within the myocardium of patients exhibiting rheumatoid arthritis (505). A full proteomic investigation was undertaken by Fert-Bober et al. (506) comprehensively identifying citrullinated proteins in myofilament proteins. Protein citrullination may play an important role in the development of cardiac inflammation and fibrosis (505, 507, 508) and has been associated with an increased inflammatory cytokine and chemokine expression in autoimmune diseases, thereby promoting and contributing to cellular stress (509–511). Citrullination has additionally been implicated within increased expression of extracellular matrix proteins, thereby playing a role in the development of cardiac fibrosis (512–515).

Citrullination has been additionally implicated as a potential biomarker for CVD in recent years (516, 517). One of the main research directions in this area has been the role of citrullination within neutrophil extracellular traps, which have long been shown to play a role in the development of abdominal aortic aneurysms (518). There has been an adequate increase in citrullination publications over the recent years; however, its use as a clinically relevant biomarker remains elusive. Further research within this space is warranted, and technological spectrometric advancements for unambiguous identification of citrullination PTMs will aid in establishing citrullination as a biomarker for CVD.

### 7.6. Other Emerging PTMs

The focus on metabolic syndrome and diabetes has cast a light on additional PTMs that should be investigated. Lysine residues are a particular hot spot for which there are increasing numbers of PTMs identified, many of which are linked to a dysregulation of metabolism. This includes propionylation, butylation, crotonylation, malonylation, succinylation, glutarylation, β-hydroxybutylation, 2-hydroxyisobutyrylation, lactylation (519), and benzoylation (K_Bz_) (520). In the context of advanced glycation end-products (AGEs) and their potential novel functional modulation, MS-based analyses have determined extensive glycation of cardiac troponin (Tn) subunits (521). However, Tn is not the only modified myofilament protein that could alter myocyte contractility, but also myosin binding protein C (522). Another AGE-PTM that is very understudied is methylglyoxal, which has been shown to impact MI outcomes in mice (522). In skinned human and mouse cardiomyocytes, acute methylglyoxal treatment depressed both calcium sensitivity and maximal calcium-activated force in a dose-dependent manner and was correlated to a number of methylglyoxalate sarcomeric proteins (523). These are exciting research areas; however, the challenge of linking a PTM to its functional outcome is daunting, and there are dire needs for large-scale perturbation studies.

## 8. FUTURE PERSPECTIVES

Proteomics is the study of proteins and the chemical diversity achieved by genetic variants, splice variants, PTMs, proteostasis, protein dynamics, protein interacting partners, cell localization, and cell and state specificity. To achieve these diverse experimental requirements, proteomic science comprises many technologies that can be deployed and used synergistically to address a particular query. Over the last 25 years, proteomics has undergone remarkable strides with respect to study design, data acquisition, data analysis, and data sharing; each of these will continue to innovate. Improvements have been made in preparative and automated workflows, analytical separation techniques, and detection platforms, including spectrometers, immunoassays, as well as other emerging platforms. MS is a central technology that in the last few years has undergone enormous improvement in repeatability, speed, specificity, and sensitivity. These advances will enable scalable systems that are increasingly amenable to the small and diverse samples inherent to basic research, allowing larger-scale clinical research with respect to both body fluids and cell-based assays. Automated preparation platforms combined with more powerful detection platforms will increasingly provide the flexibility to balance higher throughput with an increase in proteome depth and accuracy. Along with MS, other proteomic technologies that are particularly well suited for large scale studies are capture based, such as the use of a single aptamer as a capture reagent (e.g., SomaScan (SomaLogic; Refs. 524, 525) or sandwich ELISAs that employ two different antibodies per protein (e.g., Olink; Ref. 526), which are pushing the boundaries of sample numbers, particularly in body fluids. Both SomaScan and Olink capture-based technologies aspire to build extensive assay libraries that use their merging of nucleotide detection with protein recognition to quantify the proteome. For the most part, these technologies have not yet pushed into PTMs or tissue analysis. However, it is not much of a leap to consider using a subset of these reagents for tissue spatial proteomics in a manner similar to that carried out by the large-scale academic initiative the Human Protein Atlas (funded primarily by the Knut and Alice Wallenberg Foundation) and their remarkable open-source resource database (https://www.proteinatlas.org) (328). The aim of the Human Protein Atlas has been to map the human proteins in cells, tissues, organs, and blood, using integration of various omics technologies, including antibody-based imaging, MS-based proteomics, transcriptomics, proximity extension assay (PEA)-based protein profiling (the basis of Olink technology), and systems biology.

Certainly, the growth in the area of spatial proteomics will provide greater insights into the context of cardiac cells and will provide new insights into the interactions and regional diversity of myocytes, fibroblasts, and immune cell types that comprise the healthy and diseased heart. There are now even newer innovative technologies around protein sequencing using nanopores, and readers of each specific amino acid or short sequences are being developed. These could aid in moving toward full de novo protein sequencing with tandem identification of PTMs (527). As of today, these technologies have not yet scaled to where a large number of proteins can be analyzed simultaneously, and none has been applied to questions of the heart.

The remarkable increase in sensitivity and robustness of the MS instrument in the last 3 years coupled with the ability to minimize sample requirements has led to a new era of single-cell proteomics (SCP), which was deemed unfeasible only until recently. Concerning the heart, the isolation of single cells from tissues or cell clusters is not trivial, but steady progress has paved the way (88) to finding LC-MS methods that are sufficiently robust and provide the throughput required for SCP (528). In Ref. 528, we show that individual cardiomyocytes can be isolated from human and mouse hearts and then separated with a specialized flow sorter, unbiased with respect to cell size (CellenOne); ∼96 cells can be analyzed per day by LC-MS (on the TimsTOF SCP; Bruker), with >1,000 protein IDs obtainable from individual cardiomyocytes, with the ability to cover a dynamic range comparable to bulk samples. With a similar LC-MS configuration, single skeletal muscle fibers were also able to be analyzed, making this approach a quick and feasible way to reliably separate type 1 and 2A fibers (354). Another remarkable study utilized top-down MS to define PTM forms of myofilament proteins within single muscle fibers (355). These studies show that single-cell and single-muscle fiber high-throughput proteomics will be commonplace before long. It is expected that single-cell/-muscle fiber proteomics can inform us about biological and spatial heterogeneity between the same cells across a tissue and how different regions of the heart respond to and accommodate disease. Challenges (the same as with single-cell RNAseq) remain with respect to the validation of the heterogeneity and assignment to heart location or underlying cell state.

With advances in top-down proteomics that allow for the characterization of higher-molecular weight proteins from ever more complex samples, a more wholistic picture is emerging that encompasses protein complexes along with the interplay of many PTMs. In parallel, molecular characterization platforms that provide protein-protein interactions, showcase biomolecular neighborhoods, and portray the impact of confirmational changes will be increasingly relevant to an integrated understanding of cardiac biology. Here, technologies like X-ray crystallography, cryo-electron microscopy, nuclear magnetic resonance spectroscopy, along with MS will increasingly work in concert to allow an integrated study of challenging membrane proteins, glycosylated proteins, intrinsically disordered proteins, and protein complexes.

Today, MS characterization of membrane proteins remains challenging. In the heart/cardiomyocytes there are a number of essential and well-studied ion channels that are present at an extremely low copy per cell. Our data shows that single cardiomyocytes contain many integral membrane or associated membrane proteins like RyR1, ATP2A2, KCNA4, GJA1, and PLN, as well as several mitochondrial translated transmembrane proteins (e.g., MTCO1, MTCO2, mt-ND), which are consistently identified (Chazarin B, Binek A, Karlsteadt A, Haghani A, Kreimer S, Van Eyk JE, unpublished data). However, ion channels like KCNA5, KCNJ11, and KCN2 and KCN3 are not in the linear detectable range with the discovery LC-MS methods. One potential approach would be to use targeted LC-MS assays that only quantify peptides comprised of amino acids unique to the target ion channel. Here, a multiplex targeted assay where many proteins are involved in a single assay would be useful to employ.

Beyond the proteome, an integrated multiomics view of human biology remains a technical and computational challenge. Although proteomic, transcriptomic, and metabolomic changes are increasingly quantifiable, their relationship to biological phenotypes is often less clear. These omics can provide insight into the regulation and/or function of the proteome changes. Proteomics is increasingly well positioned to help surmount the challenge of bridging omics datasets with functional biology. Yet there are many other aspects of proteomics that are available, appropriate, and yet to be applied to important questions in cardiac biology. Methods to quantify newly synthesized proteins or their protein turnover in both cells or animals (529) may provide insights into the aging and proteostasis of cells (530, 531). Identification of protein complexes in vivo will not be confined to only using targeted approaches like BioID and APEX to determine functional differences between protein isoforms (532, 533) but also have the ability for the simultaneous in vivo labeling of hundreds of protein complexes (e.g., Refs. 534, 535). Imaging and simultaneous quantifying of confirmational changes that alter protein-protein interactions on a full proteome level in vivo are quickly becoming feasible. However, the vast number of PTMs is increasing, and we yet have identified and determined the functional implications of most. PTMs account for most of the protein chemical diversity, and there have been estimated to exist close to 1 million proteoforms, 90% of which are deemed to be due to PTM alterations (536). Currently, there are many types of PTMs, already over 400! However, there are still many new PTM tools that need to be developed and/or applied to answer interesting questions pertaining to the heart (32, 537).

## 9. CONCLUSIONS

The proteome is vast, but it need not be overwhelming. Although the proteome is full of complexity, it can be studied with an expanding array of tools and technologies. These tools need to be funded and developed, and once a method is standardized it must be deployed globally in multiple settings to expand our collective knowledge. Cardiac proteomics already provides an increasingly accessible lens through which to understand the comprehensive connectivity of the cells, tissues, and structures of the heart. It forces one to consider new cellular changes, signaling paradigms, dynamic interactions, and rapidly converging workflows that can inform actionable and timely clinical treatment strategies. The many anticipated advances will inevitably rely on faster and more transparent computer software geared not only toward QC, proteoform, spatial, and structural analyses but also toward a more accessible interpretation of experimental results that is more easily integrated with a growing body of scientific knowledge. All the above points toward a faster, deeper, and broader coverage of the cardiac proteome, further increasing throughput and reducing costs. These advantages will further realize the democratization of proteomics, an ambition suggested by John Yates III in 2005 (538). To quote: “What we need is to ensure that the cutting-edge technological developments in proteomics laboratories disseminate to all levels of the research community. What we need, in short, is the democratization of MS.” This vision will increasingly encompass other technologies, allowing the field to further probe the complex biology underlying the heart.

## GRANTS

The authors are funded by The Smidt Heart Institution, Cedars-Sinai Medical Center and Foundation for the National Institutes of Health (Grant Nos. U01 DK124019-01, R01HL11136, U54CA260591-01, and R01 HL155346-01).

## DISCLOSURES

No conflicts of interest, financial or otherwise, are declared by the authors.

## AUTHOR CONTRIBUTIONS

O.A.K., K.R., B.C., L.A., C.I.M., and J.E.V. prepared figures; O.A.K., A.S., K.R., B.C., L.A., C.I.M., and J.E.V. drafted manuscript; O.A.K., A.S., K.R., B.C., L.A., C.I.M., and J.E.V. edited and revised manuscript; O.A.K., A.S., K.R., B.C., L.A., C.I.M., and J.E.V. approved final version of manuscript.
